# Molecular Mass and Localization of α-1,3-Glucan in Cell Wall Control the Degree of Hyphal Aggregation in Liquid Culture of *Aspergillus nidulans*

**DOI:** 10.3389/fmicb.2018.02623

**Published:** 2018-11-06

**Authors:** Ken Miyazawa, Akira Yoshimi, Shin Kasahara, Asumi Sugahara, Ami Koizumi, Shigekazu Yano, Satoshi Kimura, Tadahisa Iwata, Motoaki Sano, Keietsu Abe

**Affiliations:** ^1^Laboratory of Applied Microbiology, Department of Microbial Biotechnology, Graduate School of Agricultural Science, Tohoku University, Sendai, Japan; ^2^ABE-Project, New Industry Creation Hatchery Center, Tohoku University, Sendai, Japan; ^3^Department of Environmental Sciences, School of Food, Agricultural and Environmental Sciences, Miyagi University, Taiwa, Japan; ^4^Department of Biochemical Engineering, Graduate School of Engineering, Yamagata University, Yonezawa, Japan; ^5^Department of Biomaterial Sciences, Graduate School of Agricultural and Life Sciences, The University of Tokyo, Tokyo, Japan; ^6^Department of Plant and Environmental New Resources, College of Life Sciences, Kyung Hee University, Seoul, South Korea; ^7^Genome Biotechnology Laboratory, Kanazawa Institute of Technology, Hakusan, Japan; ^8^Laboratory of Microbial Resources, Department of Microbial Biotechnology, Graduate School of Agricultural Science, Tohoku University, Sendai, Japan

**Keywords:** cell wall, α-1, 3-glucan, *Aspergillus nidulans*, hyphal aggregations, alkali-soluble glucan

## Abstract

α-1,3-Glucan is one of the main polysaccharides in the cell wall of filamentous fungi. *Aspergillus nidulans* has two α-1,3-glucan synthase genes, *agsA* and *agsB*. We previously revealed that AgsB is a major α-1,3-glucan synthase in vegetative hyphae, but the function of AgsA remained unknown because of its low expression level and lack of phenotypic alteration upon gene disruption. To clarify the role of α-1,3-glucan in hyphal aggregation, we constructed strains overexpressing *agsA* (*agsA^OE^*) or *agsB* (*agsB^OE^*), in which the other α-1,3-glucan synthase gene was disrupted. In liquid culture, the wild-type and *agsB^OE^* strains formed tightly aggregated hyphal pellets, whereas *agsA^OE^* hyphae aggregated weakly. We analyzed the chemical properties of cell wall α-1,3-glucan from the *agsA^OE^* and *agsB^OE^* strains. The peak molecular mass of α-1,3-glucan from the *agsA^OE^* strain (1,480 ± 80 kDa) was much larger than that from the wild type (147 ± 52 kDa) and *agsB^OE^* (372 ± 47 kDa); however, the peak molecular mass of repeating subunits in α-1,3-glucan was almost the same (after Smith degradation: *agsA^OE^*, 41.6 ± 5.8 kDa; *agsB^OE^*, 38.3 ± 3.0 kDa). We also analyzed localization of α-1,3-glucan in the cell wall of the two strains by fluorescent labeling with α-1,3-glucan-binding domain*–*fused GFP (AGBD-GFP). α-1,3-Glucan of the *agsB^OE^* cells was clearly located in the outermost layer, whereas weak labeling was detected in the *agsA^OE^* cells. However, the *agsA^OE^* cells treated with β-1,3-glucanase were clearly labeled with AGBD-GFP. These observations suggest that β-1,3-glucan covered most of α-1,3-glucan synthesized by AgsA, although a small amount of α-1,3-glucan was still present in the outer layer. We also constructed a strain with disruption of the *amyG* gene, which encodes an intracellular α-amylase that synthesizes α-1,4-glucooligosaccharide as a primer for α-1,3-glucan biosynthesis. In this strain, the hyphal pellets and peak molecular mass of α-1,3-glucan (94.5 ± 1.4 kDa) were smaller than in the wild-type strain, and α-1,3-glucan was still labeled with AGBD-GFP in the outermost layer. Overall, these results suggest that hyphal pellet formation depends on the molecular mass and spatial localization of α-1,3-glucan as well as the amount of α-1,3-glucan in the cell wall of *A. nidulans*.

## Introduction

The fungal cell wall, which is composed mainly of polysaccharides, defines cell shape and shields cells from environmental stresses (Latgé, 2010; Yoshimi et al., 2016). In filamentous fungi, several polysaccharide components (α-glucans, β-glucans, galactomannan, and chitin) are needed for proper cell wall architecture. Some filamentous fungi secrete extracellular matrix, which also includes polysaccharides such as galactosaminogalactan (Lee and Sheppard, 2016; Yoshimi et al., 2016).

Cell wall polysaccharides of the pathogenic filamentous fungus *Aspergillus fumigatus* have been fractionated into alkali-soluble (AS) and alkali-insoluble (AI) fractions (Fontaine et al., 2000). The AS fraction contains mainly α-1,3-glucan with interconnecting α-1,4-linkage, and some galactomannan (Bernard and Latgé, 2001; Latgé, 2010), and the AI fraction is composed of chitin, β-1,6-branched β-1,3-glucan, and galactomannan (Fontaine et al., 2000; Bernard and Latgé, 2001). The alkali solubility method has been applied to fractionate cell wall components of the model filamentous fungus *A. nidulans* (Yoshimi et al., 2013, 2015) and industrial fungus *A. oryzae* (Miyazawa et al., 2016; Zhang et al., 2017b); the components of polysaccharides in both fractions derived from the two fungi are similar to those derived from *A. fumigatus* (Fontaine et al., 2000; Bernard and Latgé, 2001).

The role of α-1,3-glucan in pathogenesis and hyphal adhesion has been reported in *A. fumigatus*, *A. nidulans*, and *A. oryzae* (Beauvais et al., 2005, 2013; Maubon et al., 2006; Fontaine et al., 2010; Henry et al., 2012; Yoshimi et al., 2013; Miyazawa et al., 2016; Zhang et al., 2017b). In *A. fumigatus* and the pathogenic dimorphic yeast *Histoplasma capsulatum*, pathogen-associated molecular patterns (β-1,3-glucan and chitin) are covered by α-1,3-glucan, which prevents fungal cell recognition by host cells and promotes infection (Rappleye et al., 2004, 2007; Beauvais et al., 2013). In the rice blast fungus *Magnaporthe grisea*, α-1,3-glucan masks β-1,3-glucan and chitin and protects the fungal cell wall from digestive enzymes produced by plant cells during infection. Therefore, α-1,3-glucan acts in this fungus as a stealth factor that prevents recognition by the host (Fujikawa et al., 2009, 2012). β-1,3-Glucan and chitin are essential for maintaining cell wall integrity (Muszkieta et al., 2014; Dichtl et al., 2015). In *A. fumigatus*, a mutation in the only β-1,3-glucan synthase gene *fks1* drastically attenuates growth, and increases branching and cell lysis (Dichtl et al., 2015), which is similar to the phenotype of cells treated by caspofungin that is a β-1,3-glucan synthase inhibitor. The family 1 chitin synthase mutants Δ*chsG* and Δ*chsA*/*C*/*B*/*G* of *A. fumigatus* show reduced growth and altered mycelial morphotype (Muszkieta et al., 2014). In the family 2 chitin synthase mutant Δ*csmA*/*csmB*/*F*/*D* of *A. fumigatus*, chitin content in the cell wall was almost unaffected, but the cell wall structure of the mycelia is disorganized (Muszkieta et al., 2014).

*Aspergillus fumigatus* has three α-1,3-glucan synthase genes (*AGS1–3*), and disruptants lacking single genes or all the three genes (Δ*ags*) have been constructed (Beauvais et al., 2005; Maubon et al., 2006). The Δ*ags* strain lacked α-1,3-glucan and was less pathogenic than the parental strain. α-1,3-Glucan of *A. fumigatus* has a role in the aggregation of germinating conidia (Fontaine et al., 2010). The industrial fungus *A. niger* has five α-1,3-glucan synthase genes (*agsA–E*), and the expression of *agsA* and *agsE* is up-regulated in the presence of cell wall stress–inducing compounds such as calcofluor white and caspofungin (Damveld et al., 2005). Among the three α-1,3-glucan synthase genes of *A. oryzae* (*agsA–C*), *agsB* (orthologous to *A. nidulans agsB*) acts as the main α-1,3-glucan synthase gene (Zhang et al., 2017b). The triple disruption strain lacks α-1,3-glucan in the cell wall and forms smaller hyphal pellets than the parental strain does in liquid culture (Miyazawa et al., 2016). The triple disruptant has greater biomass and enzyme productivity than the parental strain, suggesting that the use of α-1,3-glucan-deficient strains of filamentous fungi would have improved productivity in the fermentation industry (Miyazawa et al., 2016).

Chemical structure of cell wall α-1,3-glucan in fungi was previously analyzed in fission yeast *Schizosaccharomyces pombe* (Grün et al., 2005) and *A. wentii* (Choma et al., 2013). α-Glucan from *S. pombe* consists of two interconnected linear chains (subunits, <120 residues each) of 1,3-linked α-glucose and some 1,4-linked α-glucose residues at their reducing ends as spacers (Grün et al., 2005). Alkali-soluble glucan from the cell wall of *A. wentii* consists of 25 subunits (200 residues each) of α-1,3-glucan separated by a short spacer of 1,4-linked α-glucose residues (Choma et al., 2013).

*Aspergillus nidulans* has two α-1,3-glucan synthase genes, *agsA* and *agsB*. α-1,3-Glucan in vegetative hyphae is synthesized mainly by AgsB (Yoshimi et al., 2013; He et al., 2014). The disruption of the *agsB* gene leads to the loss of α-1,3-glucan; therefore, AgsB is required for α-1,3-glucan biosynthesis under normal growth conditions (Yoshimi et al., 2013). In liquid culture, the *agsB* disruptant has fully dispersed hyphae, whereas the wild-type strain forms hyphal pellets (Yoshimi et al., 2013), suggesting that α-1,3-glucan is a hyphal aggregation factor. The *agsA* gene seems to be related to conidiation (He et al., 2014). However, the details of the *agsA* function and the chemical structure of polysaccharides synthesized by AgsA and AgsB remain unclear. In *A. nidulans*, the intracellular α-amylase *amyG* is crucial for α-1,3-glucan synthesis, whereas overexpression of the GPI-anchored α-amylase *amyD* decreases the amount of cell wall α-1,3-glucan (He et al., 2014). In the present study, we constructed the *agsA^OE^* or *agsB^OE^* strains, which overexpressed either *agsA* or *agsB* under the control of a constitutive promoter in the genetic background of *agsB* or *agsA* disruptants, respectively. The alkali-soluble glucan in the cell wall of these strains is composed of polysaccharides synthesized only by either AgsA or AgsB. In liquid culture, the abnormal hyphal dispersion of the *agsB* disruption strain was restored in the *agsA^OE^* strain, which formed hyphal pellets, suggesting that AgsA produces adhesive polysaccharides. The phenotypes of the hyphal pellets were obviously different between the *agsA^OE^* and *agsB^OE^* strains. We hypothesized that this difference is attributable to the difference in the chemical structure and/or the spatial localization in the cell wall of polysaccharides synthesized by AgsA or AgsB. In this study, we analyzed the chemical structure and localization of α-1,3-glucan in the *agsA^OE^* and *agsB^OE^* strains.

## Materials and Methods

### Strains and Growth Media

Strains are listed in Supplementary Table S1. Czapek–Dox (CD) medium was used for standard culture (Fujioka et al., 2007). To fulfill the auxotrophic requirements of *A. nidulans*, 200 μg/mL arginine, 0.02 μg/mL biotin, 0.5 μg/mL pyridoxine, 1.22 mg/mL uridine, and 1.12 mg/mL of uracil were added to the CD medium. For harvesting conidia and formation of fruiting body on plates, CD medium was supplemented with 1% glucose and 1.5% agar. To determine growth characteristics in liquid culture and analyze cell wall components, CD liquid medium was supplemented with 2% glucose.

### Construction of Plasmids and Strains

The sequences of all primers are listed in Supplementary Table S2. The fusions were performed with an In-Fusion HD Cloning Kit (Clontech Laboratories, Inc., Mountain View, CA, United States) according to the manufacturer’s instructions.

#### Disruption of the *agsA* Gene

The *agsA* gene was disrupted by using the Cre/loxP marker recycling system (Zhang et al., 2017a). To disrupt the *agsA* gene, the plasmids pAP-cre and pA-agsALR were first constructed (Supplementary Figures S1A,B). For pAP-cre construction (Supplementary Figure S1A), a fragment containing the *lox71*, *xynG2* promoter (P*xynG2*) and *Cre* (amplicon 1) was amplified from the plasmid pAAG-cre (Zhang et al., 2017a). The *pyrG* marker (amplicon 2) was amplified from *A. oryzae* genomic DNA. The two amplicons and *Bam*HI-digested pUC19 vector were fused by using an In-Fusion HD Cloning Kit (Clontech Laboratories, Inc., Mountain View, CA, United States) according to the manufacturer’s instructions. For pA-agsALR construction (Supplementary Figure S1B), the 5′ non-coding region (amplicon 3) and the coding region (amplicon 4) of the *agsA* gene were amplified from *A. nidulans* ABPU1 genomic DNA. The two amplicons and *Bam*HI-digested pUC19 vector were fused. PCR amplification was then performed with primers ANagsALR-F and ANagsALR-R from pA-agsALR as a template; the resulting fragment was fused to the fragment containing P*xynG2*, *pyrG* marker gene, and *loxP*, which was obtained by digesting pAP-cre with *Not*I (Supplementary Figure S1C). The resulting plasmid was digested with *Eco*RI and transformed into the ABPU1 (Δ*ligD*) and Δ*agsB* (Δ*ligD*) strains (Supplementary Figure S1D). Candidate strains were selected on CD medium without uridine and uracil and then cultured on CD medium with uridine and uracil containing 1% xylose to induce *Cre* expression. Strains that required uridine and uracil were isolated by culture on CD plates with or without uridine and uracil. Replacement of the *agsA* gene was confirmed by PCR (Supplementary Figure S1E).

#### Construction of the *agsA*- and *agsB*-Overexpressing Strains *agsA^OE^* and *agsB^OE^*

**FIGURE 1:**
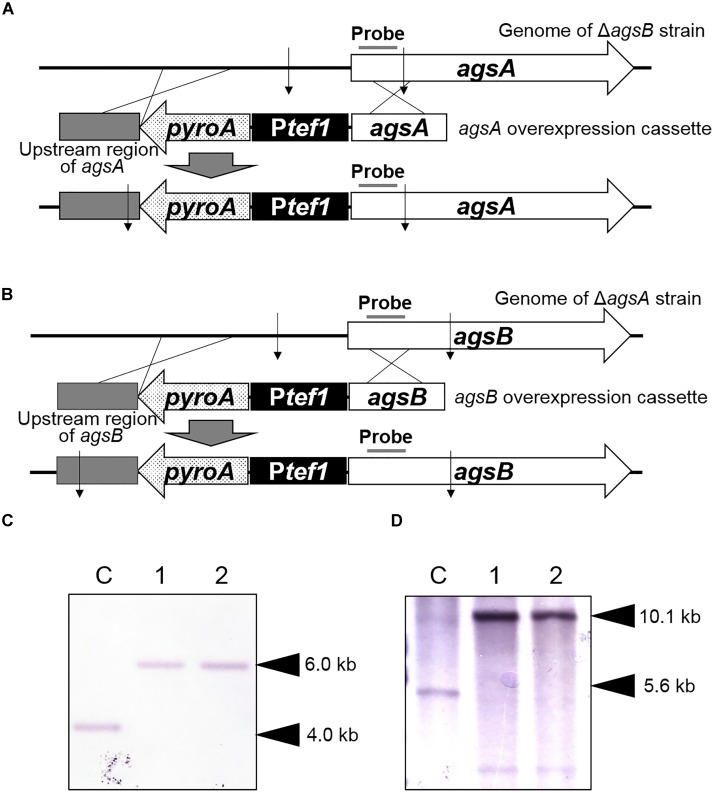
Construction of *agsA^OE^* and *agsB^OE^* strains of *Aspergillus nidulans*. **(A)** The *agsA*-overexpressing strain. Thin arrows indicate *Eco*RI digestion sites. **(B)** The *agsB* overexpressing strain. Thin arrows indicate *Sph*I digestion sites. **(C)** Southern blot analysis of the *agsA* locus with the probe indicated in **(A)**. Chromosomal DNA of the Δ*agsB* strain (lane C) and two *agsA^OE^* strains (lanes 1 and 2) was digested with *Eco*RI. **(D)** Southern blot analysis of the *agsB* locus with the probe indicated in **(B)**. Chromosomal DNA of the Δ*agsA* strain (lane C) and two *agsB^OE^* strains (lanes 1 and 2) was digested with *Sph*I.

In the *agsA^OE^* and *agsB^OE^* strains, native promoters were replaced by the constitutive *tef1* promoter. The sequences of primers used are listed in Supplementary Table S2. For generation of the *agsA^OE^* strain, the pAPyT-agsA plasmid was constructed (Supplementary Figure S2A). The 5′ non-coding region (amplicon 1) and the coding region (amplicon 2) of the *agsA* gene were amplified from *A. nidulans* ABPU1 genomic DNA. The *pyroA* marker (amplicon 3) was amplified from the pUCpyroA plasmid (Yoshimi et al., 2013). The *tef1* promoter (amplicon 4) was amplified from *A. nidulans* genomic DNA. The four amplicons and *Bam*HI-digested pUC19 vector were fused. The resulting plasmid was digested with *Not*I and transformed into the Δ*agsB* strain (Figure 1A).

For generation of the *agsB^OE^* strain, the pAPyT-agsB plasmid was constructed (Supplementary Figure S2B). The 5′ non-coding region (amplicon 1) and the coding region (amplicon 2) of the *agsB* gene were amplified from the *A. nidulans* ABPU1 genomic DNA template. The *pyroA* marker (amplicon 3) and *tef1* promoter (amplicon 4) were amplified as above. The four amplicons and *Bam*HI-digested pUC19 vector were fused. The resulting plasmid was digested with *Not*I and transformed into the Δ*agsA* strain (Figure 1B). The *agsA*- and *agsB*-overexpressing strains were isolated on the basis of *pyroA* nutrient requirement. Correct integration of each cassette was confirmed by Southern blot analysis. Genomic DNA of the Δ*agsB* and *agsA^OE^* strains was digested with *Eco*RI and that of the Δ*agsA* and *agsB^OE^* strains was digested with *Sph*I. Fragments were separated on agarose gels and transferred onto membranes, which were then hybridized with digoxigenin (DIG)-labeled specific probes (Figure 1A). As expected, the probe for *agsA* (Figure 1A) bound to a 4.0-kb fragment (Δ*agsB* strain) and a 6.0-kb fragment (*agsA^OE^* strain; Figure 1C), and the probe for *agsA* (Figure 1B) bound to a 5.6-kb fragment (Δ*agsA* strain) and a 10.1-kb fragment (*agsB^OE^* strain; Figure 1D).

Strains that required arginine were complemented by transforming them with *Bam*HI-digested pA-AoargB. This plasmid was constructed as follows (Supplementary Figure S3A). The 5′ (amplicon 1) and 3′ non-coding (amplicon 2) regions of the *argB* gene of *A. nidulans* were amplified from *A. nidulans* ABPU1 genomic DNA. The *A. oryzae argB* marker (amplicon 3) was amplified from *A. oryzae* genomic DNA. The three amplicons and *Bam*HI-digested pUC19 vector were fused. The resulting plasmid (pA-AoargB) was digested with *Not*I and transformed into arginine-requiring strains (Supplementary Figure S3B). Replacement of the *A. oryzae argB* marker was confirmed by PCR (Supplementary Figure S3C).

All strains with disrupted *ligD* were complemented with the *ligD* gene by transforming them with *Not*I-digested pAP-CligD. This plasmid was constructed as follows (Supplementary Figure S4A). The 5′ (amplicon 1) and 3′ non-coding (amplicon 2) regions of the *amdS* gene were amplified from *A. nidulans* ABPU1 genomic DNA. The *pyrG* marker (amplicon 3) was amplified from *A. oryzae* genomic DNA. The full-length *ligD* gene (amplicon 4), including its promoter and terminator regions, was amplified from *A. nidulans* ABPU1 genomic DNA. The four amplicons and *Bam*HI-digested pUC19 vector were fused. The resulting plasmid (pAP-CligD) was digested with *Not*I and transformed into the *ligD*-disrupted strains (Supplementary Figure S4B). Replacement of the *ligD* gene was confirmed by PCR (Supplementary Figure S4C).

#### Construction of *agsA* and *agsB* Promoter–GFP Reporter Strains

The strains were generated by fusing the *EGFP* gene to the 3′ end of the *agsA* or *agsB* promoter region (Supplementary Figure S5A). For generation of the *agsA* promoter–GFP reporter strain, the pUC/AoargB-PagsA-EGFP plasmid was constructed (Supplementary Figure S5). The *argB* marker gene from *A. oryzae* (amplicon 1) was amplified from *A. oryzae* genomic DNA. The 5′ non-coding region of *agsA* (amplicon 2) was amplified from *A. nidulans* ABPU1 genomic DNA, and EGFP-T*agdA* (amplicon 3) from pNA(N)EGFP (Furukawa et al., 2007). The three amplicons and *Bam*HI-digested pUC19 vector were fused. The resulting plasmid was transformed into the ABPU1 strain.

For generation of the *agsB* promoter–GFP reporter strain, the pUC/AoargB-PagsB-EGFP plasmid was constructed (Supplementary Table S1). The amplicons 1 and 3 were the same as for the construction of pUC/AoargB-PagsA–EGFP. The 5′ non-coding region of the *agsB* gene (amplicon 2) was amplified from *A. nidulans* ABPU1 genomic DNA. The three amplicons and *Bam*HI-digested pUC19 vector were fused. The resulting plasmid was transformed into the ABPU1 strain. Insertion of the cassettes was confirmed by PCR.

#### Construction of an *amyG* Gene Disruption Strain

The first round of PCR amplified gene fragments containing the 5′ non-coding region (amplicon 1) and the coding region (amplicon 2) of *amyG* from ABPU1 genomic DNA, and the *pyrG* gene (amplicon 3) from *A. oryzae* genomic DNA (Supplementary Figure S6A). The three resulting PCR products were gel-purified and fused into a disruption cassette in the second round of PCR. The resulting PCR product was gel-purified and transformed into the ABPU1 (Δ*ligD*) strain. Replacement of the *amyG* gene was confirmed by PCR (Supplementary Figure S6B).

### RNA Extraction and Quantitative PCR

Mycelial cells cultured in CD liquid medium for 24 h were collected, and total RNA was extracted from the cells by using Sepasol-RNA I (Nakalai Tesque, Kyoto, Japan) in accordance with the manufacturer’s instructions. The total RNA (2 μg) was reverse transcribed by using a High-Capacity cDNA Reverse Transcription Kit (Applied Biosystems Inc., Foster City, CA, United States). Quantitative PCR was performed with a Mini Opticon real-time PCR system (Bio-Rad Laboratories, Hercules, CA, United States) with SYBR Green detection. For reaction mixture preparation, KOD SYBR qPCR Mix (Toyobo Co., Ltd., Osaka, Japan) was used. Primers used for quantitative PCR are listed in Supplementary Table S2. An equivalent amount of cDNA, obtained from reverse transcription reactions using an equivalent amount of total RNA, was applied to each reaction mixture. The gene encoding histone H2B was used as a normalization reference (an internal control) for determining the target gene expression ratios.

### Cell Wall Fractionation

Mycelia cultured for 24 h in CD medium were collected by filtering through Miracloth (Merck Millipore, Darmstadt, Germany), washed with water, and freeze-dried. The mycelia were then pulverized by using a MM400 bench-top mixer mill (Retsch, Haan, Germany), and the powder (1 g) was suspended in 40 mL of 0.1 M Na phosphate buffer (pH 7.0). Cell wall components were fractionated by hot-water and alkali treatments (Yoshimi et al., 2013). Briefly, fractionation resulted in hot-water-soluble (HW), AS, and AI fractions; the AS fraction was further separated into a fraction soluble in water at neutral pH (AS1) and an insoluble fraction (AS2). Monosaccharide composition of each fraction was quantified according to Yoshimi et al. (2013). Each fraction was crude, and contained polysaccharides and some other compounds. For instance, the AS2 fraction derived from the wild-type strain was consisted of approximately 35% (weight) of polysaccharides (mainly α-1,3-glucan), 40% of polar lipids (<Mw 1,000) saponified by alkaline, and 25% of unidentified compounds (Yamashita et al., unpublished results).

The ABPU1 (Δ*ligD*) and *amyG* disruption strains were cultured for 16 h or 24 h in CD medium, and the mycelia were used for fractionation.

### Quantification of Liberated Sugar in Alkali-Soluble Fractions Treated With α-1,3-Glucanase

The AS2 fraction from each strain was treated with α-1,3-glucanase from *Bacillus circulans* KA-304 (Yano et al., 2006) and the amount of sugar liberated was quantified as the amount of glucose, as described by Yoshimi et al. (2013). Mutan (positive control) was enzymatically synthesized using α-1,3-glucanosyl transferase of *Streptococcus mutans* (Suyotha et al., 2013). Curdlan (β-1,3-glucan) from *Agrobacterium* spp. (Kirin Kyowa Food Co. Ltd., Tokyo, Japan) was used as a negative control.

### ^13^C NMR Analysis of the Alkali-Soluble Fraction

The AS2 fraction of each strain (50 mg) was suspended in 1 mL of 1 M NaOH/D_2_O and dissolved by vortexing. Mutan enzymatically synthesized by the action of GTF-J from *Streptococcus salivarius* (Puanglek et al., 2016) was used as a standard molecule of α-1,3-glucan. One drop of DMSO-d_6_ (deuterated dimethyl sulfoxide) was then added to each fraction and the solutions were centrifuged (3,000 × *g*, 5 min). ^13^C NMR spectra of the supernatants were obtained using a JNM-ECX400P spectrometer (JEOL, Tokyo, Japan) at 400 MHz at 35°C. Chemical shifts were recorded relative to the resonance of DMSO-d_6_.

### Methylation Analysis of Polysaccharides in the Alkali-Soluble Fraction

Methylation analysis was performed according to Ciucanu and Kerek (1984) with modifications. The AS2 fraction (3 mg) from each strain was suspended in 2 mL of 20 mg/mL NaOH in DMSO, 0.8 mL of iodomethane was added, and the mixture was stirred for 30 min at room temperature in a closed vial. Methylation was stopped by adding water (4 mL), and then chloroform (3 mL) was added. The contents of the vial were mixed, and the aqueous phase was removed. Then water (4 mL) was added to the mixture and the aqueous phase was removed; this procedure was repeated twice. Toluene (0.5 mL) was added to the chloroform phase; the mixture was dried down, resuspended in toluene (1 mL), and dried down again. The whole methylation procedure was then repeated.

90% (w/v) Formic acid (2 mL) was added to the dried methylated material; the solution was then incubated in boiling water for 3 h and dried down. Subsequently, 1 M trifluoroacetic acid (2 mL) was added to the dried material, and the sample was incubated in boiling water for 3 h and then dried down. The hydrolyzed sample was mixed with NaBH_4_ in water (33 mg/mL; 1 mL) and incubated for 16 h at room temperature. Methanol–acetic acid mixture (5 μL acetic acid in 1 mL of methanol) was added to the reduced sample, and the mixture was evaporated to dryness. Methanol–acetic acid was added again to the dried material and the mixture was dried down; this procedure was performed three times. Methanol (3 mL) was added to the dried material, and the mixture was dried down; this procedure was performed three times. Pyridine (0.6 mL) and acetic anhydride (0.6 mL) were added to the dried material, and the sample was incubated for 90 min at 90°C. Toluene (2 mL) was then added and the sample was dried down. Toluene (2 mL) was added again to the dried material and then dried down. The sample was mixed with chloroform (2 mL) and water (4 mL), and the aqueous phase was removed. Then water (4 mL) was mixed with the chloroform phase, and the aqueous phase was removed. Then toluene (0.5 mL) was added to the chloroform phase, and the mixture was dried down. Finally, the sample was mixed with chloroform (3 mL). Methylated sugars were analyzed using a gas chromatography system (GC-2010/GCMS-QP2010; Shimadzu, Kyoto, Japan) with an HP-5 column (Agilent Technologies, Inc., Santa Clara, CA, United States). Peaks were identified by comparison with information in the Shimadzu mass spectral libraries.

### Determination of the Average Molecular Mass of Polysaccharides

The average molecular mass of alkali-soluble polysaccharides was determined by high-performance size-exclusion chromatography (HPSEC). The HPSEC system consisted of an isocratic pump Dionex ICS-5000 (Dionex, Sunnyvale, CA, United States) and an FLC-10 refractive index detector (Shimamura Tech Inc., Tokyo, Japan) and was equipped with a Sugar KS-805 column (8.0 mm × 300 mm; Showa Denko Co. Ltd., Tokyo, Japan) and a Sugar KS-G 6B guard column (Showa Denko). The eluent was 1 M NaOH, and the flow rate was 1 mL/min for 25 min. The AS2 fraction (10 mg) was dissolved in 1 M NaOH. The detector was normalized with dextran standard (2,160, 1,400, 500, 110, 70, 40, 20, and 10 kDa). HY-PCR software (Shimamura Tech Inc.) was used for data analysis.

### Smith Degradation

The sample (20 mg) was suspended in 0.1 M acetate buffer (pH 3.9; 10 mL) and sodium periodate was added (final conc. 30 mM). The mixture was incubated for 72 h at 4°C in the dark with stirring. Ethylene glycol (100 μL) was then added to remove excess sodium periodate. The sample was dialyzed against water, mixed with NaBH_4_ (30 mg), and incubated for 3 h at room temperature. The sample was neutralized with 10% (v/v) acetic acid and dialyzed against water. Trifluoroacetic acid was then added (final conc. 0.5 M) and the mixture was incubated for 24 h at room temperature. The sample was freeze-dried and then dissolved in 1 M NaOH for HPSEC analysis.

### Fluorescent Labeling of Cell Wall Polysaccharides

Mycelial cells were cultured at 37°C for 12 h in CD liquid medium. A drop of cells was placed on a glass slide and dried at 55°C for 15 min. The cells were washed twice with phosphate buffered saline (PBS), fixed with 4% (v/v) paraformaldehyde for 15 min, and then washed again as above. A drop of PBS containing 100 μg/mL α-1,3-glucan-binding domain*–*fused GFP (AGBD-GFP; DS1CB6SD2-GFP; Suyotha et al., 2013), 100 μg/mL monoclonal β-1,3-glucan-specific antibody (Biosupplies, Parkville, VIC, Australia), and 10 μg/mL wheat germ agglutinin Alexa Fluor 350 conjugate was added. The sample was incubated at room temperature for 3 h and then washed as above. A drop of PBS containing anti-mouse IgG antibody Alexa Fluor 594 conjugate (Invitrogen, Carlsbad, CA, United States) as a secondary antibody for β-1,3-glucan-specific antibody was added. The sample was incubated at room temperature for 1 h, washed as above, and imaged on a FluoView FV1000 confocal laser scanning microscope (Olympus, Tokyo, Japan). For enzymatic digestion of α-1,3-glucan, fixed cells were washed twice with 50 mM potassium phosphate buffer (pH 6.5), incubated in the same buffer containing 50 μg/mL α-1,3-glucanase from *B. circulans* KA-304 (Yano et al., 2006) for 6 h at 37°C, washed three times with PBS, and fluorescently labeled. For enzymatic digestion of β-1,3-glucan, fixed cells were washed twice with 50 mM sodium acetate buffer (pH 5.0), incubated in the same buffer containing 500 μg/mL β-1,3-glucanase from *A. niger* (Sigma, St. Louis, MO, United States) for 6 h at 55°C, washed three times with PBS, and fluorescently labeled.

### α-1,3-Glucan Detection by Using α-1,3-Glucan-Binding Domain–Fused GFP

To detect α-1,3-glucan in the cell wall of vegetative hyphae and fruiting body, cells were stained with AGBD-GFP. Cells (20 mg) of vegetative hyphae or fruiting body of the BPU1 strain (Hagiwara et al., 2007) were suspended in 50 mM potassium phosphate buffer (pH 6.5) containing 0.1 mg/mL AGBD-GFP and incubated at 30°C for 4 h with gentle shaking. The cells were washed twice with 50 mM potassium phosphate buffer (pH 6.5) and observed under an IX81 inverted fluorescence microscope (Olympus).

### Statistical Analysis

Student’s *t*-test was used for the comparison of paired samples, and Tukey’s test was used for the comparison of multiple samples.

## Results

### Characterization of *agsA^OE^* and *agsB^OE^* Strains in Liquid Culture

**FIGURE 2:**
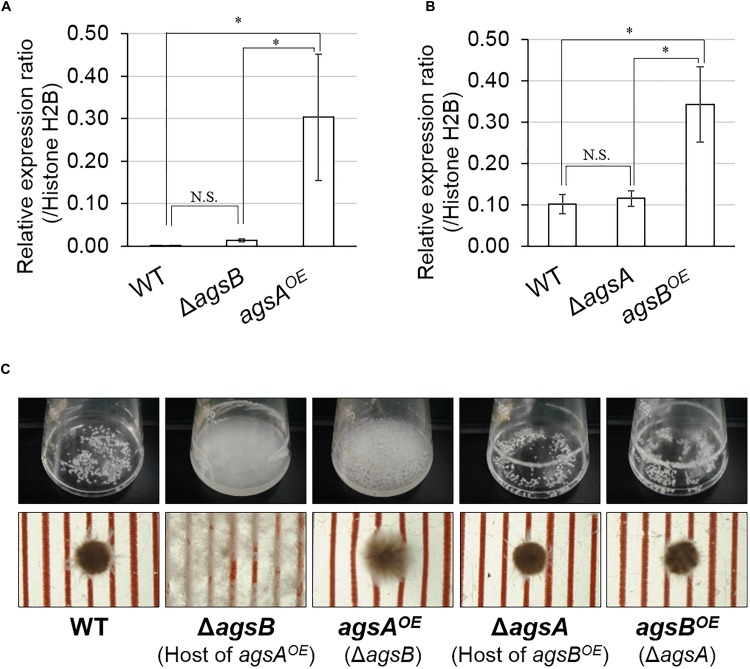
Characterization of the *agsA^OE^* and *agsB^OE^* strains in liquid culture. Conidia (final concentration, 5 × 10^5^/mL) of the wild-type, *agsA^OE^*, *agsB^OE^*, Δ*agsA*, and Δ*agsB* strains were inoculated into CD liquid medium and rotated at 160 rpm at 37°C for 24 h. **(A,B)** Transcript levels of the **(A)** *agsA* and **(B)** *agsB* genes were determined by quantitative PCR on total RNA using the gene-specific primers indicated in Supplementary Table S2. Error bars represent the standard deviation of the mean calculated from three replicates. Asterisks denote significant differences based on Tukey’s test (^∗^*P* < 0.05). **(C)** Growth of the wild-type, Δ*agsB*, *agsA^OE^*, Δ*agsB*, *agsB^OE^* strains. Upper row, photographs of cultures in Erlenmeyer flasks; lower row, representative hyphal pellets of each strain under a stereomicroscope. Scale intervals are 1 mm.

We constructed the *A. nidulans* strains each overexpressing only one α-1,3-glucan synthase gene by introducing an *agsA* overexpression cassette into the Δ*agsB* strain and *agsB* cassette into the Δ*agsA* strain. In hyphal cells, the expression level of *agsA* was significantly higher in the *agsA^OE^* strain than in the wild-type and Δ*agsB* strains (*P* < 0.05; Figure 2A). The expression of *agsB* was higher in the *agsB^OE^* strain than in the wild-type and Δ*agsA* strains (*P* < 0.05; Figure 2B). The results of the quantitative PCR analysis also revealed that *agsA* in the *agsB^OE^* strain and *agsB* in the *agsA^OE^* strain were not expressed (data not shown). Thus, the expression of *agsA* or *agsB* was upregulated in the respective strains.

No distinguishable differences were observed in the colonial growth and conidiation of any of the strains grown on agar plates for 4 days (Supplementary Figure S7). In liquid culture, the hyphae of the wild-type and Δ*agsA* strains formed tightly aggregated pellets, whereas the Δ*agsB* strain showed dispersed hyphae (Figure 2C), in agreement with our previous results (Yoshimi et al., 2013). The phenotype of hyphal dispersion of the Δ*agsB* strain was restored in the *agsA^OE^* strain, but the hyphae of the *agsA^OE^* strain formed loosely aggregated pellets (Figure 2C). The hyphae of the *agsB^OE^* strain formed tightly aggregated pellets, similar to those of the wild type (Figure 2C). These results suggest that the phenotypic difference between the *agsA^OE^* and *agsB^OE^* strains might be attributable to the difference in polysaccharides synthesized by AgsA and AgsB.

### Overexpression of *agsA* or *agsB* Increases the Amount of Alkali-Soluble Glucan

**FIGURE 3:**
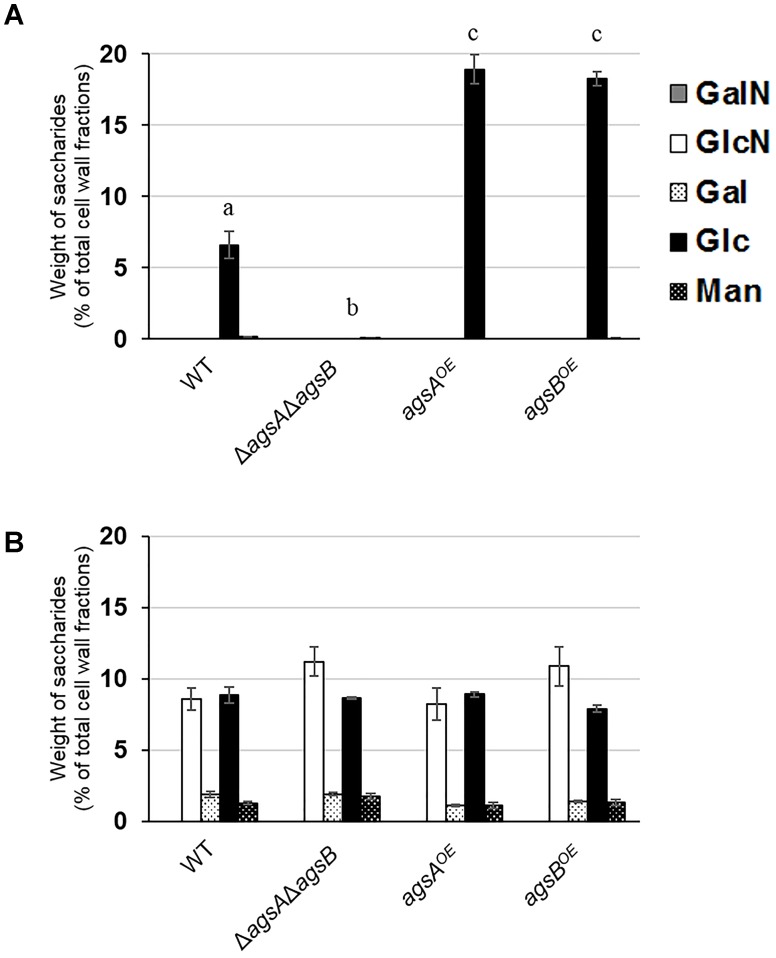
Monosaccharide composition of cell wall fractions from the wild-type (WT), Δ*agsA*Δ*agsB*, *agsA^OE^*, and *agsB^OE^* strains. The strains were cultured in CD medium (160 rpm, 37°C, 24 h). **(A)** Alkali-soluble, insoluble in water at neutral pH fraction (AS2). **(B)** Alkali-insoluble (AI) fraction. Error bars represent the standard error of the mean calculated from three replicates. Different letters denote significant differences based on Tukey’s test (*P* < 0.05). GalN, galactosamine; GlcN, glucosamine; Gal, galactose; Glc, glucose; Man, mannose.

To analyze cell wall components in the *agsA^OE^* and *agsB^OE^* strains, we fractionated the cell walls of both strains by hot-water and alkali treatments (Yoshimi et al., 2013). The HW fraction contained mainly glucose (Glc) with small amounts of galactose (Gal) and mannose (Man) (Supplementary Figure S8A). The amount of glucose in the HW fractions of the *agsA^OE^* and *agsB^OE^* strains was lower than in those of the wild-type strain (Supplementary Figure S8A). The AS1 fraction contained little sugar (Supplementary Figure S8B). AS2 was composed mainly of Glc with a small amount of Man, although AS2 of the Δ*agsA*Δ*agsB* strain hardly contained any Glc (Figure 3A). The amount of Glc in the AS2 fraction from both the *agsA^OE^* (18.9% of total cell wall fractions) and *agsB^OE^* (18.2%) strains was about three times that of the wild type (6.6%). The AI fraction was composed of about 10% of glucosamine and Glc with 1–2% Gal and Man in each strain (Figure 3B). Taken together, these results show that the replacement of the native *agsA* or *agsB* promoter with the *tef1* promoter increased the amount of alkali-soluble glucan in the cell wall of both the *agsA^OE^* and *agsB^OE^* strains.

### Alkali-Soluble Glucan From Both the *agsA^OE^* and *agsB^OE^* Strains Is Composed Mainly of α-1,3-Glucan

**FIGURE 4:**
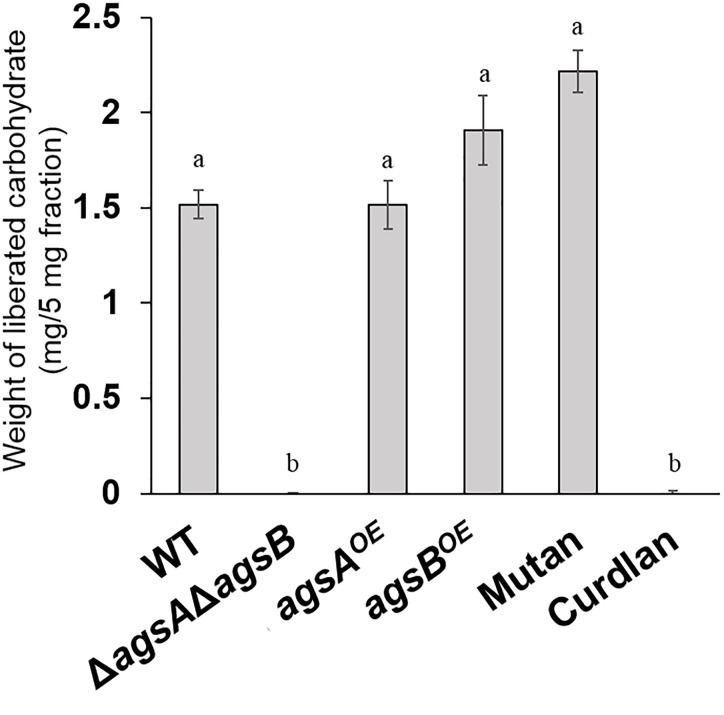
Saccharides liberated from the AS2 fractions after treatment with α-1,3-glucanase. The AS2 fractions derived from the wild-type, Δ*agsA*Δ*agsB*, *agsA^OE^*, and *agsB^OE^* strains were treated with α-1,3-glucanase from *Bacillus circulans* KA-304. Liberated sugars were hydrolyzed and quantified as glucose by high-performance liquid chromatography. Error bars represent the standard error of the mean calculated from three replicates. Different letters denote significant differences based on Tukey’s test (*P* < 0.05).

Our previous analyses showed that the AS2 fraction derived from wild-type *A. nidulans* contains mainly α-1,3-glucan (Yoshimi et al., 2013). To investigate the factor that caused the difference in hyphal aggregation between the *agsA^OE^* and *agsB^OE^* strains, we analyzed the polysaccharide composition of the AS2 fraction. Sugar liberated from the AS2 fraction by α-1,3-glucanase was detected in the wild-type strain and the positive control mutan but was scarce in the AS2 fraction from the Δ*agsA*Δ*agsB* strain and the negative control curdlan (Figure 4). In the AS2 fractions from both the *agsA^OE^* and *agsB^OE^* strains, sugar was liberated similar to that from the wild-type strain and mutan (Figure 4).

**FIGURE 5:**
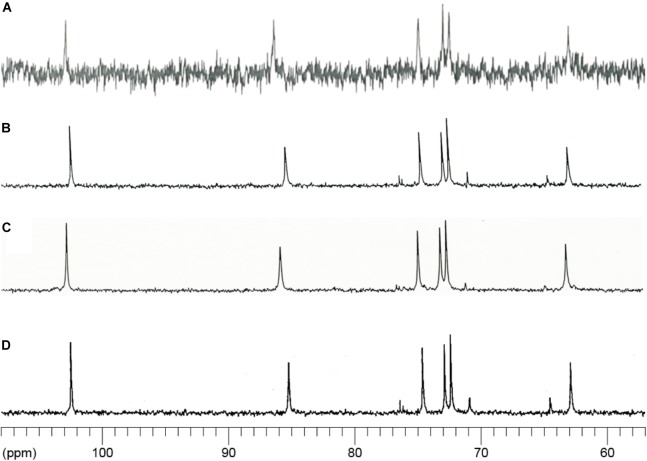
^13^C-NMR spectra of the AS2 fractions from the wild-type (WT), *agsA^OE^*, and *agsB^OE^* strains. The AS2 fractions from **(A)** wild-type, **(B)** *agsA^OE^*, and **(C)** *agsB^OE^* strains were dissolved in 1 M NaOH/D_2_O. **(D)** Mutan enzymatically synthesized by the action of GTF-J from *Streptococcus salivarius* and representing a standard molecule of α-1,3-glucan. NMR spectra were measured at 400 MHz at 35°C. Chemical shifts were recorded relative to the resonance of DMSO-d_6_.

In agreement with Yoshimi et al. (2013), ^13^C NMR spectroscopic analysis detected signals typical of α-1,3-glucan in the AS2 fraction from the wild-type strain and in mutan, which was enzymatically synthesized by GTF-J from *S. salivarius* (Figures 5A,D). The AS2 fractions from both the *agsA^OE^* and *agsB^OE^* strains also showed chemical shift signals similar to those of mutan (Figures 5B,C).

To determine the linkage of polysaccharides in the AS2 fraction, we performed methylation analysis. The AS2 fraction from the wild-type strain contained mainly 2,4,6-tri-*O*-methyl(Me)-Glc (1,3,5-triacetyl-2,4,6-tri-*O*-methyl-D-glucitol), which indicated 1,3-glucosidic bonds (Table 1). Small amounts of 2,3,4,6-tetra-*O*-Me-Glc (indicating non-reducing end termini), 2,3,6-tri-*O*-Me-Glc (1,4-glucosidic bonds), 3,4,6-tri-*O*-Me-Glc (1,2-glucosidic bonds), and 2,4-di-*O*-Me-Glc (3,6-branching points) were also detected in the AS2 fraction from the wild-type strain (Table 1). The 1,4-glucosidic bonds seemed to be derived from the primer or spacer molecules of α-1,3-glucan (Table 1). The 1,2-glucosidic bonds seemed to be derived from galactomannan (Figure 3A). The 3,6-branching points might be derived from contaminating β-1,3/1,6-glucan (Table 1). Similar to the AS2 fraction from the wild-type strain, those from the *agsA^OE^* and *agsB^OE^* strains contained the same permethylated glucose compounds as each other (Table 1). Taken together, the results of all three experimental approaches show that the AS2 fractions from the wild-type, *agsA^OE^*, and *agsB^OE^* strains were composed mainly of α-1,3-glucan.

**Table 1:** Methylation analysis of alkali-soluble glucan.

Alditol acetate	Linkage	RT	Molar amount (%)		
			
		(min)	WT	*agsA^OE^*	*agsB^OE^*
2,3,4,6-Tetra-*O*-Me-Glc	**G_1-_**	15.1	0.69	0.46	0.73
2,4,6-Tri-*O*-Me-Glc	**_-3_G_1-_**	16.4	89.31	94.09	87.98
2,3,6-Tri-*O*-Me-Glc	**_-4_G_1-_**	16.5	5.41	1.74	7.01
3,4,6-Tri-*O*-Me-Glc	**_-2_G_1-_**	17.4	2.08	1.45	1.68
2,4-Di-*O*-Me-Glc	**^-^_-_^6^_3_G_1-_**	17.9	2.51	2.26	2.42


*2,3,4,6-Tetra-O-Me-Glc, 1,5-di-acetyl-2,3,4,6-tetra-O-methyl-D-glucitol.*

*2,4,6-Tri-O-Me-Glc, 1,3,5-tri-acetyl-2,4,6-tri-O-methyl-D-glucitol*.

*2,3,6-Tri-O-Me-Glc, 1,4,5-tri-acetyl-2,3,6-tri-O-methyl-D-glucitol*.

*3,4,6-Tri-O-Me-Glc, 1,2,5-tri-acetyl-3,4,6-tri-O-methyl-D-glucitol*.

*2,4-Di-O-Me-Glc, 1,3,5,6-tetra-acetyl-2,4-di-O-methyl-D-glucitol*.

*RT, retention time.*

### Average Molecular Mass of Alkali-Soluble Glucan Is Larger in *agsA^OE^* Than in *agsB^OE^*

**FIGURE 6:**
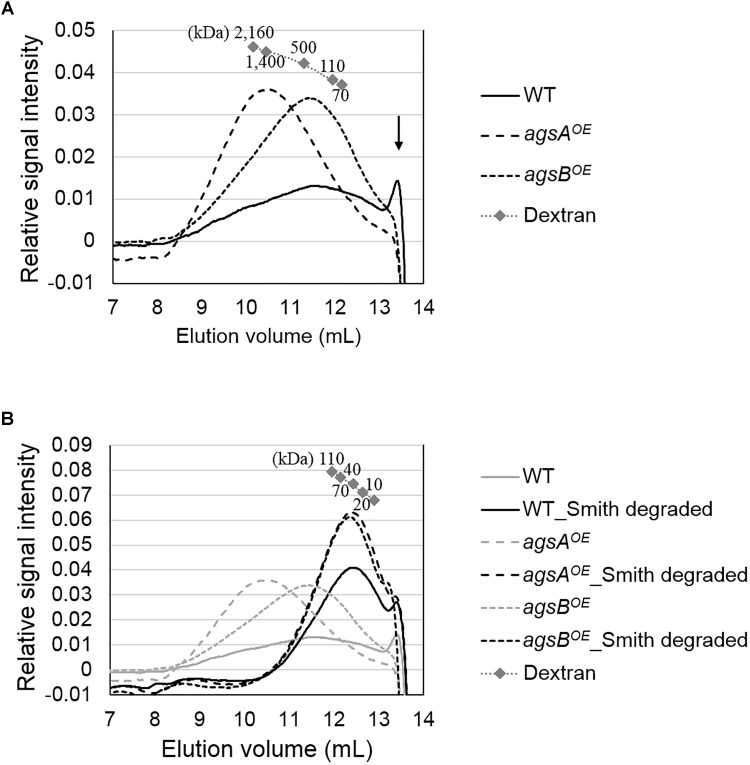
High-performance size-exclusion chromatography elution profiles of **(A)** AS2 fractions and **(B)** Smith-degraded AS2 fractions from the wild-type, *agsA^OE^*, and *agsB^OE^* strains. The AS2 fraction (10 mg) from each strain was dissolved in 1 M NaOH/H_2_O. Glucan elution was monitored as refractive index. Molecular masses of the glucan peaks were determined from a calibration curve of dextran standards. Elution profiles shown in gray in **(B)** are the same as in **(A)**. The results of a representative of three experiments are shown.

The molecular mass of alkali-soluble glucan from filamentous fungi is expected to be nearly 1,000 kDa (Choma et al., 2013) and is therefore difficult to estimate. Here, we efficiently evaluated the molecular mass of alkali-soluble glucan using HPSEC. The increase in the elution volume of dextrans (molecular weight standards for polysaccharides) with the decrease in their molecular weight could be approximated by a quadratic function in the 70-2,160 kDa range (Figure 6A). The peak of alkali-soluble glucan from the *agsA^OE^* strain was detected in a smaller elution volume than that from the *agsB^OE^* strain (Figure 6A). The peak molecular weight (*M*_p_) of the alkali-soluble glucan from the *agsA^OE^* strain was 1,480 ± 80 kDa, which was about four times that from *agsB^OE^* (372 ± 47 kDa; *P* < 0.01) (Table 2). The alkali-soluble glucan from the wild-type strain (147 ± 52 kDa) was smaller than that from the *agsB^OE^* strain (*P* < 0.05; Table 2). The amount of alkali-soluble glucan from the wild type was also smaller than that from the *agsB^OE^* stain; therefore, the alkali-soluble glucan from the wild type might be more sensitive to degradation. These molecular masses are equivalent to number-average degrees of polymerization (DP) of 9,160 ± 520 (*agsA^OE^*), 2,230 ± 290 (*agsB^OE^*), and 908 ± 319 (wild type) (Table 2). In the wild type, an additional large peak was detected at approximately 13.5 mL, which seemed to be derived from degraded alkali-soluble cell wall glucan (Figure 6A; black arrow). The difference in the molecular mass of alkali-soluble glucan between the *agsA^OE^* and *agsB^OE^* strains might be a primary factor causing hyphal aggregation.

**Table 2:** Molecular mass and degree of polymerization of alkali-soluble glucans from α-1,3-glucan synthase–overexpressing strains.

Sample	*M*_p_^a^ (kDa)	DP^b^
WT AS2^c^	147 ± 52	908 ± 319
WT AS2, Smith-degraded	31.6 ± 3.7	195 ± 23
*agsA^OE^* AS2	1,480 ± 80	9,160 ± 520
*agsA^OE^* AS2, Smith-degraded	41.6 ± 5.8	257 ± 36
*agsB^OE^* AS2	372 ± 47	2,230 ± 290
*agsB^OE^* AS2, Smith-degraded	38.3 ± 3.0	237 ± 19


*^*a*^Molecular mass of the peak.*

*^*b*^Degree of polymerization.*

*^*c*^AS2, insoluble components after dialysis of the alkali-soluble fraction. Values represent the mean ± standard deviation of three replicates.*

### Alkali-Soluble Glucans From Both the *agsA^OE^* and *agsB^OE^* Strains Consist of Similar Repeating Subunits

We hypothesized that the chemical structure such as that of α-1,3-glucan subunits might differ between alkali-soluble glucans from the *agsA^OE^* and *agsB^OE^* strains, and thus we performed Smith degradation, which selectively hydrolyzes 1,4-linked glucose residues in alkali-soluble glucan chains (Goldstein et al., 1965; Hay et al., 1965), followed by HPSEC.

Smith-degraded alkali-soluble glucans from both the *agsA^OE^* and *agsB^OE^* strains were detected in a larger elution volume than that of non-degraded glucans (Figure 6B). The molecular mass of Smith-degraded glucans was estimated by calibration using dextran (10–110 kDa) with linear approximation (Figure 6B). The *M*_p_ of Smith-degraded glucan was 41.6 ± 5.8 kDa for *agsA^OE^*, 38.3 ± 3.0 kDa for *agsB^OE^*, and 31.6 ± 3.7 kDa for the wild-type (Table 2). These *M*_p_ values are equivalent to DP of 257 ± 36 for *agsA^OE^*, 237 ± 19 for *agsB^OE^*, and 195 ± 23 for the wild type (Table 2). No significant difference was found in the degree of polymerization of Smith-degraded alkali-soluble glucan between the *agsA^OE^* and *agsB^OE^* strains. The numbers of α-1,3-glucan subunits in the alkali-soluble glucan calculated from the molecular masses of non-degraded and Smith-degraded alkali-soluble glucans were 35.9 ± 2.8 (*agsA^OE^*), 9.82 ± 2.27 (*agsB^OE^*), and 4.65 ± 1.89 (wild-type). These HPSEC analyses suggest that the molecular mass of alkali-soluble glucan synthesized by AgsA is approximately four times that synthesized by AgsB, and that AgsA and AgsB synthesize subunits consisting of similar numbers (approximately 200) of 1,3-linked α-glucose residues.

### α-1,3-Glucan Is Located in the Outer Layer of the Cell Wall in the *agsB^OE^* Strain, but in the Inner Layer in the *agsA^OE^* Strain

**FIGURE 7:**
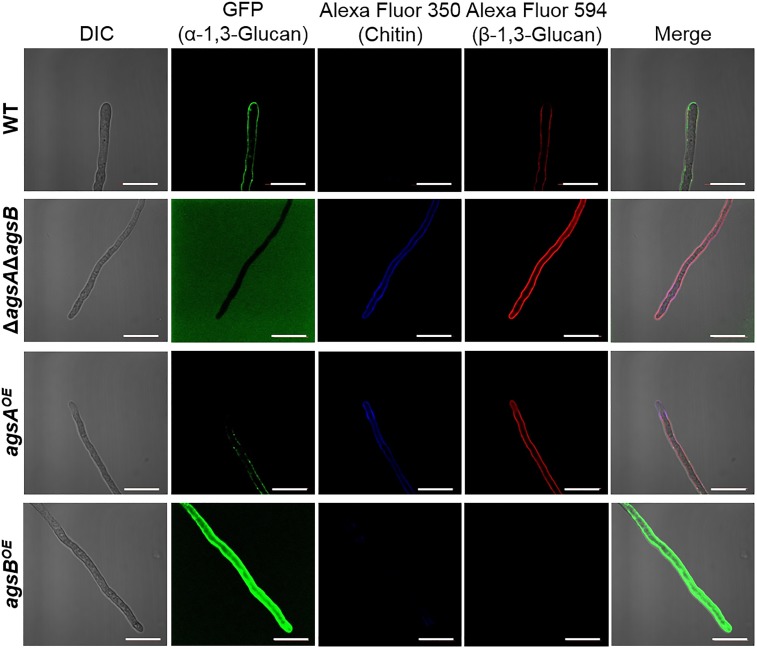
Localization of cell wall polysaccharides of vegetative hyphae. Hyphae cultured in CD liquid medium for 12 h were fixed and stained with AGBD-GFP for α-1,3-glucan, fluorophore-labeled antibody for β-1,3-glucan, and fluorophore-labeled lectin for chitin. Scale bars are 10 μm.

We found that the phenotypes of the *agsA^OE^* and *agsB^OE^* strains might be related to the molecular weight of the alkali-soluble glucan, which mainly consisted of α-1,3-glucan. However, another possibility could be that spatial localization of α-1,3-glucan synthesized by AgsA in the cell wall differs from that of AgsB. To examine this possibility, we labeled α-1,3-glucan, β-1,3-glucan, and chitin in the cell wall of the wild-type, *agsA^OE^*, and *agsB^OE^* strains. In the wild-type strain, α-1,3-glucan was strongly labeled along the outline of the cells, whereas β-1,3-glucan and chitin were labeled weakly (Figure 7). In the Δ*agsA*Δ*agsB* strain, which lacks cell wall α-1,3-glucan, β-1,3-glucan, and chitin were strongly labeled along the outline of the cells (Figure 7). In the *agsB^OE^* strain, α-1,3-glucan was strongly labeled along the outline of the cells, but it was only weakly labeled in the *agsA^OE^* strain (Figure 7). In addition, β-1,3-glucan and chitin were clearly labeled in the *agsA^OE^* strain, but not in the *agsB^OE^* strain (Figure 7). These results suggest that α-1,3-glucan of the wild-type and *agsB^OE^* strains was localized in the outermost layer of the cell wall, whereas α-1,3-glucan of the *agsA^OE^* strain might be masked by β-1,3-glucan and chitin layer.

**FIGURE 8:**
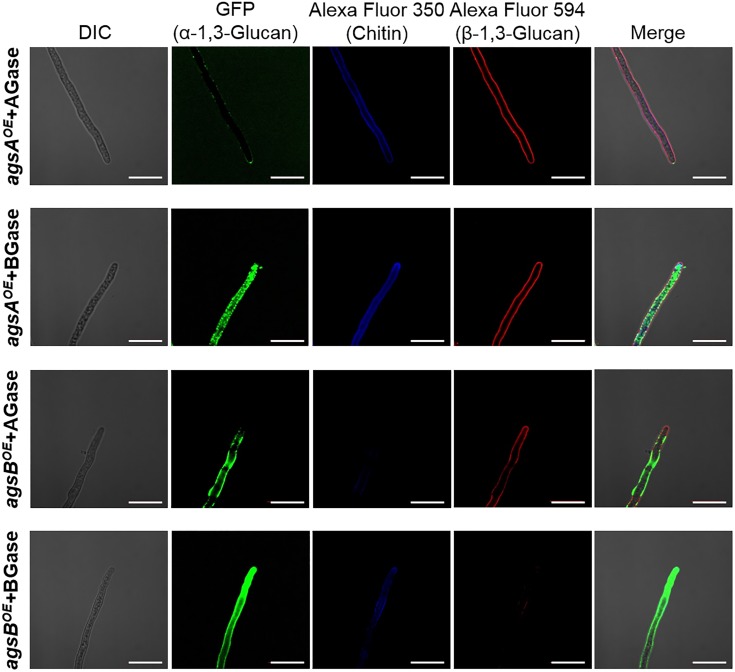
Localization of cell wall polysaccharides after treatment with α-1,3-glucanase or β-1,3-glucanase. Vegetative hyphae cultured for 12 h were fixed and treated with α-1,3-glucanase (AGase) or β-1,3-glucanase (BGase) for 6 h. The hyphae were stained with AGBD-GFP for α-1,3-glucan, fluorophore-labeled antibody for β-1,3-glucan, and fluorophore-labeled lectin for chitin. Scale bars are 10 μm.

To validate these results, we treated *agsA^OE^* and *agsB^OE^* cells with α-1,3-glucanase or β-1,3-glucanase, and then observed the fluorescent-labeled cell wall components. α-1,3-Glucanase-treated *agsA^OE^* cells and β-1,3-glucanase-treated *agsB^OE^* cells (Figure 8) looked similar to the respective untreated cells (Supplementary Figure S9). In the β-1,3-glucanase-treated *agsA^OE^* cells, α-1,3-glucan was clearly labeled at the outline of the cells with some fluorescent dots, and β-1,3-glucan and chitin labeling was also visible (Figure 8). Fluorescence of α-1,3-glucan was weaker in the α-1,3-glucanase-treated *agsB^OE^* cells (Figure 8) than in untreated *agsB^OE^* cells (Supplementary Figure S9). In contrast, fluorescence intensity of β-1,3-glucan was increased after treatment with α-1,3-glucanase in the *agsB^OE^* cells (Figure 8) compared to the untreated cells (Supplementary Figure S9). These results suggest that most α-1,3-glucan of *agsB^OE^* cells is present in the outermost layer of the cell wall and that of *agsA^OE^* cells is masked by β-1,3-glucan.

### GFP Fluorescence Is Detected in Hülle Cells in Both the P*agsA*-EGFP and P*agsB*-EGFP Strains

**FIGURE 9:**
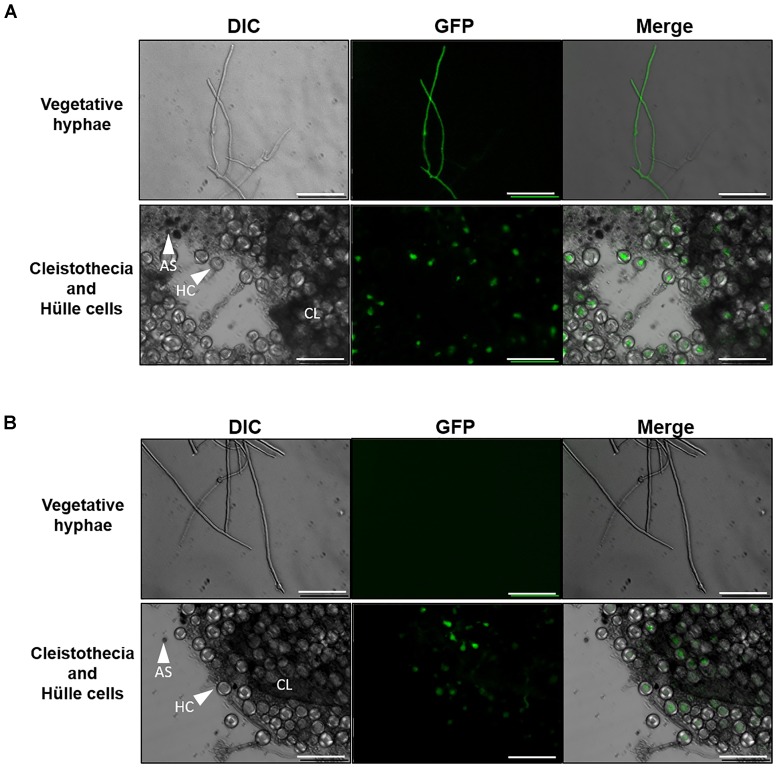
Fluorescence microscopy of *Aspergillus nidulans* P*agsA*-EGFP and P*agsB*-EGFP strains. **(A)** P*agsB*-EGFP strain. **(B)** P*agsA*-EGFP strain. Hyphae during vegetative growth (top) and a fruiting body (bottom) are shown. The vegetative hyphae were cultured in CD liquid medium at 37°C for 24 h. Fruiting bodies were obtained by culture on CD agar medium for 8 days. Scale bars are 50 μm. HC, Hülle cell; AS, ascospore; CL, cleistothecium.

We constructed *agsA*- and *agsB*-promoter GFP reporter strains (i.e., P*agsA*-EGFP, P*agsB*-EGFP) and examined the expression of these genes during sexual development. During vegetative growth, clear fluorescence was observed in the hyphae of the P*agsB*-EGFP strain (Figure 9A), but no fluorescence was detected in the P*agsA*-EGFP strain (Figure 9B), in agreement with the published data (Yoshimi et al., 2013; He et al., 2014). Interestingly, GFP signals in both the P*agsA*- and P*agsB*-EGFP strains were observed in Hülle cells (nursing cells of cleistothecia) during sexual development, but not in cleistothecia or ascospores (Figures 9A,B). These results suggest that the *agsA* and *agsB* genes are expressed in Hülle cells during sexual development.

### α-1,3-Glucan Is Detected in Hülle Cells in the BPU1 Strain by Staining With AGBD-GFP

**FIGURE 10:**
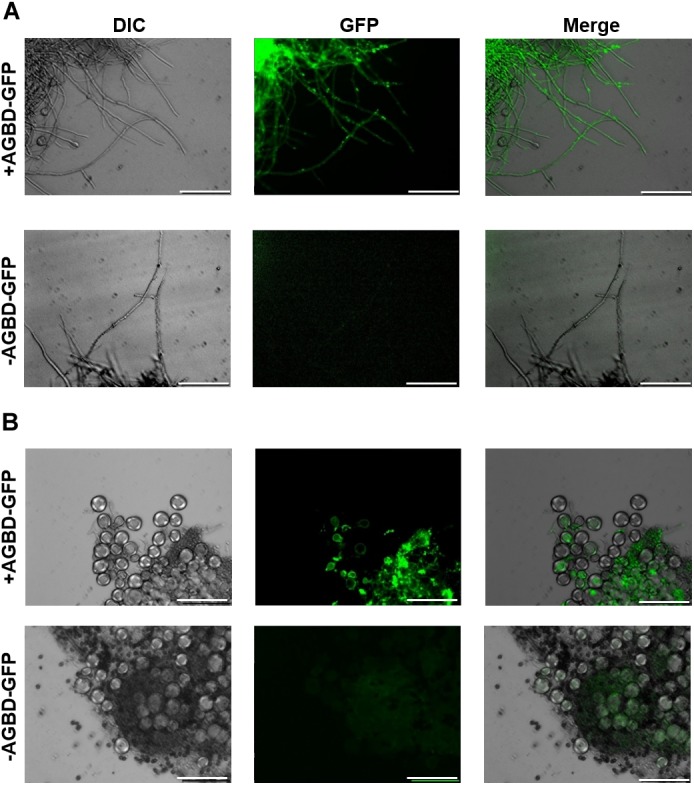
Fluorescence microscopy of the BPU1 strain stained with α-1,3-glucan-binding domain–fused GFP (AGBD-GFP). **(A)** Vegetative growth stage; **(B)** sexual development. The cells (20 mg) were stained in 50 mM potassium phosphate buffer (pH 6.5) with or without 0.1 mg/mL AGBD-GFP at 30°C for 4 h and then observed under an inverted fluorescence microscope. Scale bars are 50 μm.

Although Wei et al. (2001) reported the expression of the α-1,3-glucanase gene *mutA* in Hülle cells in *A. nidulans*, there was no direct evidence for the presence of α-1,3-glucan in these cells. To examine whether α-1,3-glucan is present in wild-type Hülle cells, we stained them with AGBD-GFP. The vegetative hyphae of the BPU1 strain were clearly stained (Figure 10A). In the fruiting body, fluorescence was observed in Hülle cells and in hyphae near the cleistothecia (Figure 10B). These results show that α-1,3-glucan is present not only in vegetative hyphae but also in the cells of the fruiting body, in particular Hülle cells.

### Small Hyphal Pellets Formed by the *amyG* Disruption Strain Are Caused by a Decreased Molecular Mass of Alkali-Soluble Glucan

**FIGURE 11:**
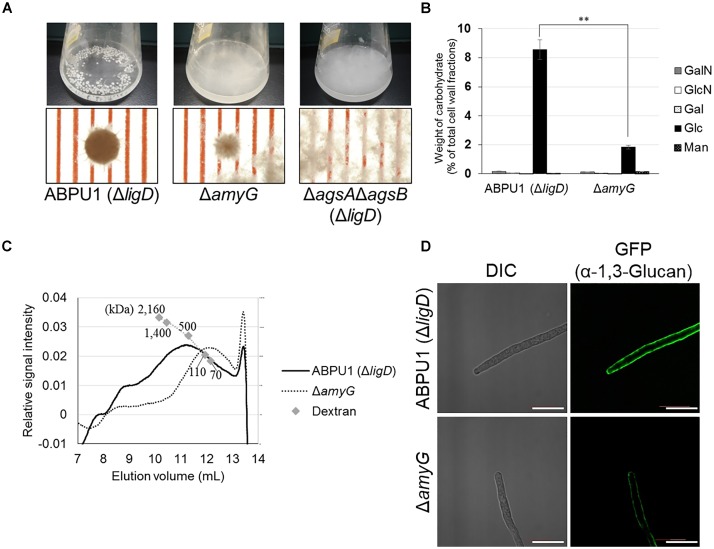
Characterization of the *amyG* disruption (Δ*amyG*) strain of *Aspergillus nidulans*. **(A)** Growth of the ABPU1 (Δ*ligD*), Δ*amyG*, and Δ*agsA*Δ*agsB* (Δ*ligD*) strains. Conidia (final concentration, 5.0 × 10^5^/mL) of each strain were inoculated into CD liquid medium and rotated at 160 rpm at 37°C for 24 h. Upper row, photographs of cultures in Erlenmeyer flasks; lower row, representative hyphal pellets under a stereomicroscope. Scale intervals are 1 mm. **(B)** Monosaccharide composition of the AS2 fractions from ABPU1 (Δ*ligD*) and Δ*amyG* obtained from mycelia cultured for 24 h. Error bars represent the standard error of the mean calculated from three replicates. Asterisks denote significant differences from ABPU1 (Δ*ligD*) based on Student’s *t*-test (^∗∗^*P* < 0.01). GalN, galactosamine; GlcN, glucosamine; Gal, galactose; Glc, glucose; Man, mannose. **(C)** Representative HPSEC elution profiles of the AS2 fractions from the Δ*amyG* and ABPU1 (Δ*ligD*) strains of three experiments. The AS2 fraction (10 mg) obtained from mycelia cultured for 16 h from each strain was dissolved in 1 M NaOH/H_2_O. Glucan elution was monitored as refractive index. Molecular masses of the glucan peaks were determined from a calibration curve of dextran standards. **(D)** Vegetative hyphae of the Δ*amyG* strain cultured in CD liquid medium for 12 h were fixed and stained with AGBD-GFP for α-1,3-glucan, fluorophore-labeled antibody for β-1,3-glucan, and fluorophore-labeled lectin for chitin. Scale bars are 10 μm.

To further elucidate the relationship between the molecular mass of alkali-soluble glucan and pellet morphology, we constructed an *amyG* disruption strain (Δ*amyG*) of *A. nidulans*. Disruption of this gene in *A. nidulans* markedly decreases the amount of cell wall α-1,3-glucan, and the disruptant forms small hyphal pellets in liquid culture (He et al., 2014). In agreement with the results of He et al. (2014), our Δ*amyG* strain formed smaller and more loosely aggregated pellets than did the parental strain ABPU1 (Δ*ligD*) (Figure 11A). The amount of glucose in the AS2 fraction derived from the Δ*amyG* strain was significantly decreased (Figure 11B). The amount of alkali-soluble glucan obtained from the mycelia of Δ*amyG* strain cultured for 24 h was markedly small (data not shown), which caused rapid degradation of α-1,3-glucan by background of α-1,3-glucanase activity in the strain. Therefore, we used mycelia cultured for 16 h to prevent α-1,3-glucan degradation for the analysis of the molecular mass of AS2 glucan, because mycelia of 16 h culture showed lower α-1,3-glucan degradation activity. Interestingly, the molecular mass of alkali-soluble glucan from the Δ*amyG* strain (93.5 ± 1.4) was significantly smaller than that from the parental strain (355 ± 165; Figure 11C and Table 3; *P* < 0.05). These results suggest that the appropriate molecular mass of alkali-soluble glucan is required for the formation of tightly aggregated hyphal pellets.

**Table 3:** Molecular mass and degree of polymerization of alkali-soluble glucan from the *amyG* disruption strain.

Sample	*M*_p_^a^ (kDa)	DP^b^
ABPU1 (Δ*ligD*) AS2	355 ± 165	2190 ± 1020
Δ*amyG* AS2	93.5 ± 1.4	577 ± 9


*^*a*^Molecular mass of the peak.*

*^*b*^Degree of polymerization.*

We also stained α-1,3-glucan with AGBD-GFP in the cell wall of the ABPU1 (Δ*ligD*) and Δ*amyG* strains. In the ABPU1 (Δ*ligD*) strain, α-1,3-glucan was clearly stained (Figure 11D), similar to that in the wild-type strain (Figure 7), whereas the labeling was weak but still detectable at the outline of the cells in the Δ*amyG* strain (Figure 11D). These results suggest that α-1,3-glucan in the Δ*amyG* strain is localized in the outermost layer of the cell wall.

## Discussion

Although the role of α-1,3-glucan in pathogenesis and hyphal adhesion has been reported in *A. fumigatus*, *A. nidulans*, and *A. oryzae* (Beauvais et al., 2005, 2013; Maubon et al., 2006; Fontaine et al., 2010; Henry et al., 2012; Yoshimi et al., 2013; Miyazawa et al., 2016; Zhang et al., 2017b), the chemical structure of α-1,3-glucan in filamentous fungi has not been analyzed, with one exception. In *A. wentii*, Choma et al. (2013) reported the chemical structure and properties of alkali-soluble glucan as a substrate for mutanase (α-1,3-glucanase), which can be used as a tooth paste ingredient to prevent the growth of *S. mutans. A. nidulans* has two α-1,3-glucan synthase genes, *agsA* and *agsB*. α-1,3-Glucan in vegetative hyphae is synthesized mainly by AgsB (Yoshimi et al., 2013; He et al., 2014). The *agsA* gene seems to be associated with conidiation (He et al., 2014). However, the details of the *agsA* function and the chemical structure of polysaccharides synthesized by AgsA and AgsB remain unclear.

**FIGURE 12:**
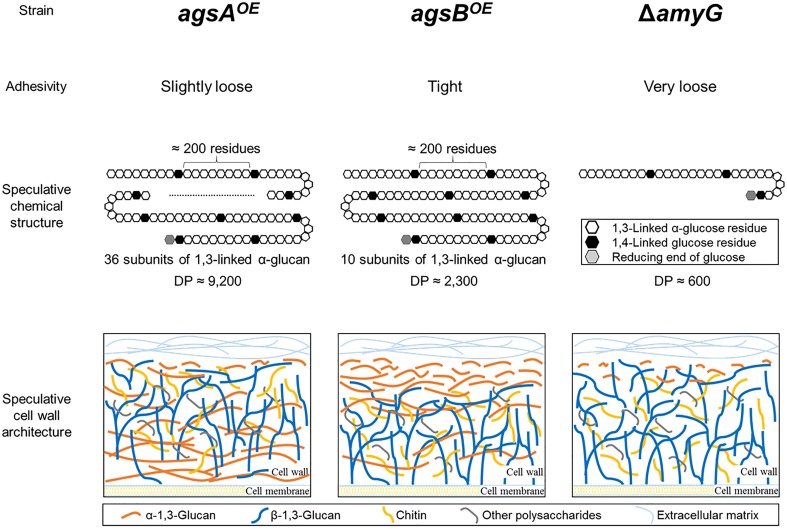
Speculative chemical structure of alkali-soluble glucans and cell wall architecture of the *agsA^OE^*, *agsB^OE^*, and Δ*amyG* strains of *A. nidulans*. The alkali-soluble glucan fraction from *A. nidulans* consists mainly of linear 1,3-linked α-glucan. 1,3-Linked α-glucan subunits (∼200 residues each) might be separated by short 1,4-linked α-glucan spacers. The degree of polymerization (DP) of the alkali-soluble glucans differ markedly among the *agsA^OE^*, *agsB^OE^*, and Δ*amyG* strains. The distribution of α-1,3-glucan in the cell wall might differ between the *agsA^OE^* (most in the inner layer and some in the outer layer) and *agsB^OE^* (outer layer) strains. In the Δ*amyG* strain, α-1,3-glucan was localized on the outermost layer of the cell wall although the amount of α-1,3-glucan was decreased.

In the present study, we constructed strains overexpressing *agsA* or *agsB* (*agsA^OE^* and *agsB^O^*^E^) and analyzed in detail the structure of polysaccharides from the cell wall of these strains. The hyphae of the *agsA^OE^* strain loosely aggregated to form pellets, whereas the hyphae of the wild-type and *agsB^OE^* strains aggregated tightly (Figure 2C). Several analyses of the chemical structure of alkali-soluble glucan revealed that alkali-soluble glucan from the wild-type, *agsA^OE^*, and *agsB^OE^* strains was mainly composed of α-1,3-glucan (Figures 4, 5 and Table 1), and the *M*_p_ values of alkali-soluble glucans from the wild-type and *agsB^OE^* strains were ∼150 and ∼370 kDa, respectively, whereas that from the *agsA^OE^* strain was 1,480 kDa (Table 2). Smith degradation of the AS2 fractions showed that alkali-soluble glucan in the *agsA^OE^*, *agsB^OE^*, and wild-type strains was composed of a number of subunits, each consisting of approximately 200 residues of 1,3-linked α-glucose (Table 2). The number of α-1,3-glucan subunits in the *agsA^OE^* strain was four times that in the *agsB^OE^* strain. The model of alkali-soluble glucan synthesized by AgsA and AgsB of *A. nidulans* is shown in Figure 12.

The reason why the molecular mass of alkali-soluble glucan differed between the *agsA^OE^* and *agsB^OE^* strains remains unclear. α-1,3-Glucan synthase Ags1 of *S. pombe* contains three functional domains: extracellular, intracellular, and a multipass transmembrane domain. Both AgsA and AgsB of *A. nidulans* are also predicted to contain these domains (He et al., 2014). 1,3-Linked α-glucan chains are predicted to be synthesized by the intracellular domain (probably with 1,4-linked α-glucan primer), and then exported outside of the cell through the transmembrane domain. Exported α-1,3-glucan chains are interconnected by the transglycosylation reaction catalyzed by the extracellular domain, resulting in mature α-1,3-glucan (Yoshimi et al., 2017). The temperature-sensitive mutant *S. pombe* strain *ags1-1^ts^*, which has the mutation G696S in the extracellular domain, synthesizes immature shortened α-glucan chains because of the probable defect in transglycosylation (Grün et al., 2005). Although G696 is conserved in both AgsA and AgsB of *A. nidulans*, the surrounding residues are not highly conserved (Miyazawa et al., unpublished data). The difference in the molecular weight of alkali-soluble glucan between the *agsA^OE^* and *agsB^OE^* strains might depend on the functional difference between the extracellular domains of the two synthases. Further biochemical and enzymatic characterization of AgsA and AgsB is necessary to understand these differences.

Fluorescence microscopy suggests that α-1,3-glucans of the wild-type and *agsB^OE^* strains are localized in the outermost layer of the cell wall, but most α-1,3-glucan of the *agsA^OE^* strain is localized in an inner layer of the cell wall (Figures 7, 8). This difference might be explained by the probable differences in (1) enzymatic properties of AgsA and AgsB, which may directly affect the spatial localization of α-1,3-glucan, and (2) physicochemical properties (such as solubility in the water phase), which may be affected by the molecular mass of α-1,3-glucan. Cell wall polysaccharides are generally synthesized at the hyphal tips during apical growth (Riquelme et al., 2018). After the biosynthesis of α-1,3-glucan on the plasma membrane, sugar chains are released to the outside of the membrane, insolubilized and immobilized to be a part of the cell wall (Latgé and Beauvais, 2014). Larger polysaccharides are usually less soluble than the smaller ones (Guo et al., 2017). Therefore, larger α-1,3-glucan molecules might be localized in the inner layer of the cell wall, whereas smaller ones might be rather distributed toward the outer layer of the cell wall (Figure 12). The proportion of low-molecular-mass alkali-soluble glucan was greater in the *agsB^OE^* cells than in the *agsA^OE^* cells (Figure 6A), which might result in more α-1,3-glucan being displayed in the outermost layer of the *agsB^OE^* cell wall (Figure 7). The weak fluorescence of AGBD-GFP observed along the outline of *agsA^OE^* cells without α-1,3-glucanase treatment (Figure 7) might be attributable to cell-surface α-1,3-glucan (with lower molecular mass values) that contributes to the loose hyphal aggregation (Figure 2C). AGBD-GFP labeling of α-1,3-glucan in the Δ*amyG* strain (Figure 11D) might be also consistent with the second possibility (Figure 12). Recently, the molecular architecture of the cell wall of *A. fumigatus* was determined by using solid-state NMR, and α-1,3-glucans were localized in the outer shell and inner domain of the cell wall (Kang et al., 2018). In wild-type *A. nidulans*, the distribution of α-1,3-glucan synthesized by the two synthases differed in the cell wall; this might depend on the molecular mass of the α-1,3-glucan. Further study of the relationship between localization and physicochemical properties (such as solubility) of α-1,3-glucan with different molecular masses is necessary.

In fungi, not only α-1,3-glucan synthases but also intracellular α-amylases and UTP-glucose-1-phosphate uridylyltransferase are involved in biosynthesis of α-1,3-glucan (Marion et al., 2006; Camacho et al., 2012; He et al., 2014). Intracellular amylases seem to synthesize α-1,4-oligoglucan primers for α-1,3-glucan synthesis (Marion et al., 2006; van der Kaaij et al., 2007; Camacho et al., 2012). The intracellular α-amylase AmyD of *A. niger* hydrolyzes starch and produces mainly maltotriose (van der Kaaij et al., 2007). Disruption of *amyG* in *A. nidulans* significantly decreases the amount of α-1,3-glucan in the cell wall (He et al., 2014). The alkali-soluble glucan in *A. wentii* contains short spacers of 1,4-linked α-glucose residues that separate α-1,3-glucan subunits (Choma et al., 2013). We determined that the alkali-soluble glucan of *A. nidulans* consists of about 90% 1,3-linked α-glucan with a small amount of Smith-degradable glucan (maybe 1,4-linked α-glucan); the subunits of α-1,3-glucan may be separated by 1,4-linked glucan (Figures 6, 12 and Table 2). The molecular mass of alkali-soluble glucan derived from the *A. nidulans* Δ*amyG* strain was 93.5 kDa, which was significantly smaller than that from the parental strain (Table 3). AmyG might synthesize the spacer structure for α-1,3-glucan. The molecular weight of Smith-degraded alkali-soluble glucan from the *A. nidulans* Δ*amyG* strain was smaller than that of non-degraded alkali-soluble glucan (our preliminary data), implying that an unknown factor involved in spacer synthesis besides AmyG is present in *A. nidulans*. Further study is needed to reveal the mechanism of α-1,3-glucan biosynthesis in filamentous fungi.

In *A. nidulans*, transcription of the α-1,3-glucan synthase genes is regulated mainly by the cell wall integrity signaling (CWIS) pathway during vegetative growth (Fujioka et al., 2007). This pathway contains a mitogen activated protein (MAP) kinase, MpkA, which regulates transcription of α-1,3-glucan synthase genes via the transcription factor RlmA (Fujioka et al., 2007). We observed the expression of *agsA* and *agsB* in Hülle cells during sexual development (Figure 9), and AGBD-GFP staining revealed that Hülle cells contained α-1,3-glucan (Figure 10). No *agsB* expression was detected in hyphal cells during sexual development (data not shown). Therefore, the expression of *agsA* and *agsB* might be regulated by a signaling pathway other than the CWIS pathway. In *A. nidulans*, the α-1,3-glucanase gene *mutA* is expressed in Hülle cells (Wei et al., 2001), the hexose transporter gene *hxtA* is expressed in Hülle cells and vegetative hyphae during carbon starvation (Wei et al., 2004), and α-1,3-glucan seems to be a storage polysaccharide for cleistothecium formation (Zonneveld, 1972). Based on these data, α-1,3-glucan might have the following temporal and spatial localization: (1) synthesis in vegetative hyphae; (2) degradation in vegetative hyphae under starvation; (3) synthesis in Hülle cells; and (4) consumption to develop cleistothecia.

Fontaine et al. (2010) reported that α-1,3-glucan has a role in aggregation of germinating conidia in *A. fumigatus*, and we reported that α-1,3-glucan in the cell wall contributes to the adhesion of *A. nidulans* hyphae in liquid culture (Yoshimi et al., 2013). Since the disruption of the *amyG* gene leads to smaller hyphal pellets than in a wild-type *A. nidulans* strain (He et al., 2014), formation of hyphal pellets is expected to depend on the amount of α-1,3-glucan. We attributed the phenotypic difference of hyphal pellets between the *agsA^OE^* and *agsB^OE^* strains in liquid culture to the difference in the molecular mass (Table 2) and localization of alkali-soluble α-1,3-glucan in the cell wall (Figures 7, 8). The *A. nidulans* Δ*amyG* strain formed smaller hyphal pellets, and the molecular mass of its α-1,3-glucan was considerably smaller than that of the wild type. α-1,3-Glucan of the Δ*amyG* strain was still labeled with AGBD-GFP. Thus, this smaller α-1,3-glucan localized in the outermost layer of the cell wall still showed weak adhesive properties, allowing the formation of hyphal pellets (Figure 11). Therefore, the shape and size of hyphal pellets seem to be controlled not only by the amount but also by the molecular mass and localization of α-1,3-glucan in the cell wall. Although we demonstrated that the two α-1,3-glucan synthases synthesize α-1,3-glucans with different molecular masses, the mechanisms by which the two enzymes regulate the molecular mass of α-1,3-glucan remain unclear. The mechanisms that regulate the spatial localization of α-1,3-glucan in the cell wall also need to be investigated in the future. Further studies to determine the molecular mechanism of α-1,3-glucan biosynthesis are underway.

## Author Contributions

KM, AY, and KA conceived and designed the experiments. KM, AY, and MS constructed fungal mutants. KM and ShK performed methylation analysis and ^13^C NMR. AS carried out the Southern blot analysis. AK and SY produced α-1,3-glucanase, mutan from *S. mutans*, and AGBD-GFP. SaK and TI produced mutan from *S. salivarius*. KM performed most experiments and analyzed the data.

## Conflict of Interest Statement

The authors declare that the research was conducted in the absence of any commercial or financial relationships that could be construed as a potential conflict of interest.
