# Supplementary material for: Molecular Mass and Localization of α-1,3-Glucan in Cell Wall Control the Degree of Hyphal Aggregation in Liquid Culture of Aspergillus nidulans
Source: Front Microbiol. 2018 Nov 6;9:2623. doi: 10.3389/fmicb.2018.02623 (PMC6232457; doi:10.3389/fmicb.2018.02623)
Supplement: Supplementary file 1 [file Data_Sheet_1.docx]

Supplementary Material

**Molecular Mass and Localization of α-1,3-Glucan in Cell Wall Control the Degree of Hyphal Aggregation in Liquid Culture of *Aspergillus* *nidulans***

**Ken Miyazawa, Akira Yoshimi, Shin Kasahara, Asumi Sugahara, Ami Koizumi, Shigekazu Yano, Satoshi Kimura, Tadahisa Iwata, Motoaki Sano, and Keietsu Abe***

*** Correspondence:** Keietsu Abe: kabe@niche.tohoku.ac.jp

**TABLE S1. Strains used in this study.**

| **Strain** | **Genotype** | **Reference** |
| --- | --- | --- |
| ABPU1 | *biA1*, *pyrG89*, *wA3*, *argB2*, *pyroA4*, *veA1* | (Motoyama et al. 1996) |
| ABPU1 (Δ*ligD*) | *biA1*, *pyrG89*, *wA3*, *argB2*, *pyroA4*, *veA1*, *ligD*::*ptrA* | (Yoshimi et al. 2013) |
| ABPU1 (*ligD*^+,^ *argB*^+^; wild-type) | *biA1*, *pyrG89*, *wA3*, *argB2*, *pyroA4*, *veA1*, *ligD*::*ptrA*, *ligD*+::*pyrG*, *AoargB*^+^ | This study |
| Δ*agsA* | *biA1*, *pyrG89*, *wA3*, *argB2*, *pyroA4*, *veA1*, *ligD*::*ptrA*, *agsA*::*loxP*, *ligD*^+^::*pyrG*, *AoargB*^+^ | This study |
| Δ*agsB* | *biA1*, *pyrG89*, *wA3*, *argB2*, *pyroA4*, *veA1*, *ligD*::*ptrA*, *agsB*::*argB*, *ligD*^+^::*pyrG* | This study |
| Δ*agsA*Δ*agsB* | *biA1, pyrG89, wA3, argB2, pyroA4, veA1, ligD::ptrA, agsA::loxP, agsB::argB, ligD^+^::pyrG, AoargB^+^* | This study |
| *agsA^OE^* | *biA1*, *pyrG89*, *wA3*, *argB2*, *pyroA4*, *veA1*, *ligD*::*ptrA*, *agsB*::*argB*, *ligD*^+^::*pyrG*, *Ptef1*-*agsA*::*pyroA* | This study |
| *agsB^OE^* | *biA1*, *pyrG89*, *wA3*, *argB2*, *pyroA4*, *veA1*, *ligD*::*ptrA*, *agsA*::*loxP*, *ligD*^+^::*pyrG*, *AoargB*^+^, *Ptef1*-*agsB*::*pyroA* | This study |
| BPU1 | *biA1*, *pyrG89*, *wA3*, *argB2*, *pyroA4*, *veA1*, *AoargB*^+^ | (Hagiwara et al. 2007) |
| P*agsA*-*EGFP* | *biA1*, *pyrG89*, *wA3*, *argB2*, *pyroA4*, *veA1*, P*agsA*-*EGFP*::*argB* | This study |
| P*agsB*-*EGFP* | *biA1*, *pyrG89*, *wA3*, *argB2*, *pyroA4*, *veA1*, P*agsB*-*EGFP*::*argB* | This study |
| Δ*amyG* | *biA1*, *pyrG89*, *wA3*, *argB2*, *pyroA4*, *veA1*, *ligD*::*ptrA*, *amyG*::*pyrG* | This study |
| Δ*agsA*Δ*agsB* (Δ*ligD*) | *biA1*, *pyrG89*, *wA3*, *argB2*, *pyroA4*, *veA1*, *ligD*::*ptrA*, *agsB*::*argB* | (Yoshimi et al. 2013) |

**TABLE S2. Primers used in this study**

| **Purpose** | **Primer name** | **Sequence (5' to 3')** |
| --- | --- | --- |
| Construction of the *agsA* gene disruption strain | | |
|  | PxynG2-cre-F | GGTACCCGGGGATCGCGGCCGCTACCGTTCGTATAGCATACA |
|  | PxynG2-cre-R | GGGTACGTCTGTTGTCCTCGTACTCGATGACGGCT |
|  | AopyrG-PU1 | ACAACAGACGTACCCTGTGATGTTC |
|  | AopyrG-PL1-lox66 | CGACTCTAGAGGATCGCGGCCGCTACCGTTCGTATAATGTATGCTATACGAAGTTATAACTGCACCTCAGAAGAA |
|  | ANagsA-LU1 | CGACTCTAGAGGATCCAGTGGAGGAGTTAGGGAGTGAT |
|  | ANagsA-LL1 | CGTAATAATCTGCTGAAAGAGTAAGGTAGAAGCCCC |
|  | ANagsA-RU1 | CAGCAGATTATTACGCACCGGA |
|  | ANagsA-RL1 | CGGTACCCGGGGATCCAACCGTGGTTTTGGTGGCAAAG |
|  | ANagsALR-F | TAAATTTCGCGGCCGCCAGCAGATTATTACGCACCGGAATG |
|  | ANagsALR-R | TAAATTTCGCGGCCGCAAAGAGTAAGGTAGAAGCCCC |
| Construction of the *agsA^OE^* and *agsB^OE^* strains | | |
|  | ANagsA-tef-LU2 | CGACTCTAGAGGATCCGCGGCCGCGACTAGTTAGCAACCAGCAAC |
|  | ANagsA-tef-LL2 | AACCCGTATACTCCTCGACTACTAGACGCTGGTTC |
|  | ANagsA-tef-RU2 | CGCACCACCTTCAAAATGAGGTGGAGGCCTTTAAACC |
|  | ANagsA-tef-RL2 | CGGTACCCGGGGATCCGCGGCCGCCTGGCCAACGAAAAACATCC |
|  | ANpyroA-PU | TGGCGCCTGTCTGGTTTGGGAAGCGCAGTTGAGCCTGAGA |
|  | ANpyroA-PL | AGGAGTATACGGGTTTTTGGCATTG |
|  | ANPtef-TU | AACTGCGCTTCCCAAACCAGACAGGCGCCACTC |
|  | ANPtef-TL | tttgaaggtggtgcgaacttt |
|  | ANagsB-tef-LU | CGACTCTAGAGGATCCGCGGCCGCGCTTCACCAACAATCCTTCCACCTG |
|  | ANagsB-tef-LL | AACCCGTATACTCCTGTGAGGCGTACCATGGGTACTG |
|  | ANagsB-tef-RU | CGCACCACCTTCAAAATGGGGAGGCTCCAGCTCTCA |
|  | ANagsB-tef-RL | CGGTACCCGGGGATCCGCGGCCGCTGGCGTTAATCAGGTCGTTGG |
| Construction of the *argB* complementation strain | | |
|  | ANargB-LU | CGACTCTAGAGGATCCGCGGCCGCACAGTTCGCTGTAAGCTCATCGGA |
|  | ANargB-LL | CAGCGGATGGAATTCAGCAGGAGCTTCCGATCATTG |
|  | ANargB-RU | TACTCCGTCGGTACCAGTCGTCCTAGCCAAGGTAGATC |
|  | ANargB-RL | CGGTACCCGGGGATCCGCGGCCGCGCACTGAAAGATCATGTGCTTGCCA |
|  | AoargB-F | CGGTACCCGGGGATCGGTACCGACGGAGTAACT |
|  | AoargB-R | GAATTCCATCCGCTGTGG |
| Construction of the *ligD* complementation strain | | |
|  | ANamdS-LU | CGACTCTAGAGGATCCGCGGCCGCCTACAACATGAGGTGTTGCCTCCTG |
|  | ANamdS-LL | GATACAATACGGATCCCGGAGGCCTCTGTGATCTTCAGTTC |
|  | ANamdS-RU | GGGTACGTCTGTTGTTAACGATAGCTCAGCCTTGCAGGTG |
|  | ANamdS-RL | CGGTACCCGGGGATCCGCGGCCGCTGTCGCCAGAGTGCTTCACTTGAAC |
|  | AopyrG-PU2 | GGGTCTCATCGGATCCAACTGCACCTCAGAAGAAAAGGATG |
|  | AopyrG-PL2 | ACAACAGACGTACCCTGTGATGTTC |
|  | ANligD-F | CATTCATGCAGGATCCGTATTGTATC |
|  | ANligD-R | ATAGAAGAGATGGATCCGATGAGACC |
| Quantitative PCR | | |
|  | ANagsA-RT-F | GCTTTCCAAATCCCACAGTTGG |
|  | ANagsA-RT-R | GTGAAGCAGATATGCATCCGTG |
|  | ANagsB-RT-F | ATCGGACACTACCTTCCCTG |
|  | ANagsB-RT-R | GACTTGGCTGACGATCAACG |
|  | ANHistone-RT-F | CACCCGGACACTGGTATCTC |
|  | ANHistone-RT-R | GAATACTTCGTAACGGCCTTGG |

**TABLE S2. Primers used in this study (continued)**

| **Purpose** | **Primer name** | | **Sequence (5' to 3')** | |
| --- | --- | --- | --- | --- |
| Construction of the P*agsA*- and P*agsB*-EGFP strains | | | | |
|  | | IF_PagsA-F | | CAGCGGATGGAATTCAGTGGAGGAGTTAGGGAGTG |
|  | | IF_PagsA-R | | GCCCTTGCTCACCATGGTCGATTTTTCCGGATGTC |
|  | | IF_EGFP-F | | ATGGTGAGCAAGGGCGAG |
|  | | IF_EGFP-TagdA-R | | CGACTCTAGAGGATCAGGCCTGCAGGAGATCCC |
|  | | IF_PagsB-F | | CAGCGGATGGAATTCCAATGAGAGCTGGAATCAGTG |
|  | | IF_PagsB-R | | GCCCTTGCTCACCATAGTAACTGATGAAGCGAGAAC |
| Construction of the *amyG* gene disruption strain | | | | |
|  | | ANamyG-LU | | TCCCTCCTGCCTGTTAGCTC |
|  | | ANamyG -LL | | CACAGGGTACGTCTGTTGTTGTGCTTTGCGGCATCTAGTC |
|  | | ANamyG -RU | | TTCTTCTGAGGTGCAGTTATGCAGTATACGGTCTTGG |
|  | | ANamyG -RL | | CGTTGCTCATTATTCTACTAGG |
|  | | ANamyG -PU | | TAGATGCCGCAAAGCACAACAACAGACGTACCCTGTGATGTTC |
|  | | ANamyG -PL | | CCAAGACCGTATACTGCATAACTGCACCTCAGAAGAAAAGGATG |
|  | | ANamyG-F | | GATGTCACCACCCTCGTATC |
|  | | ANamyG-R | | AACGTCCTTGACAATCTAGTTGCTC |


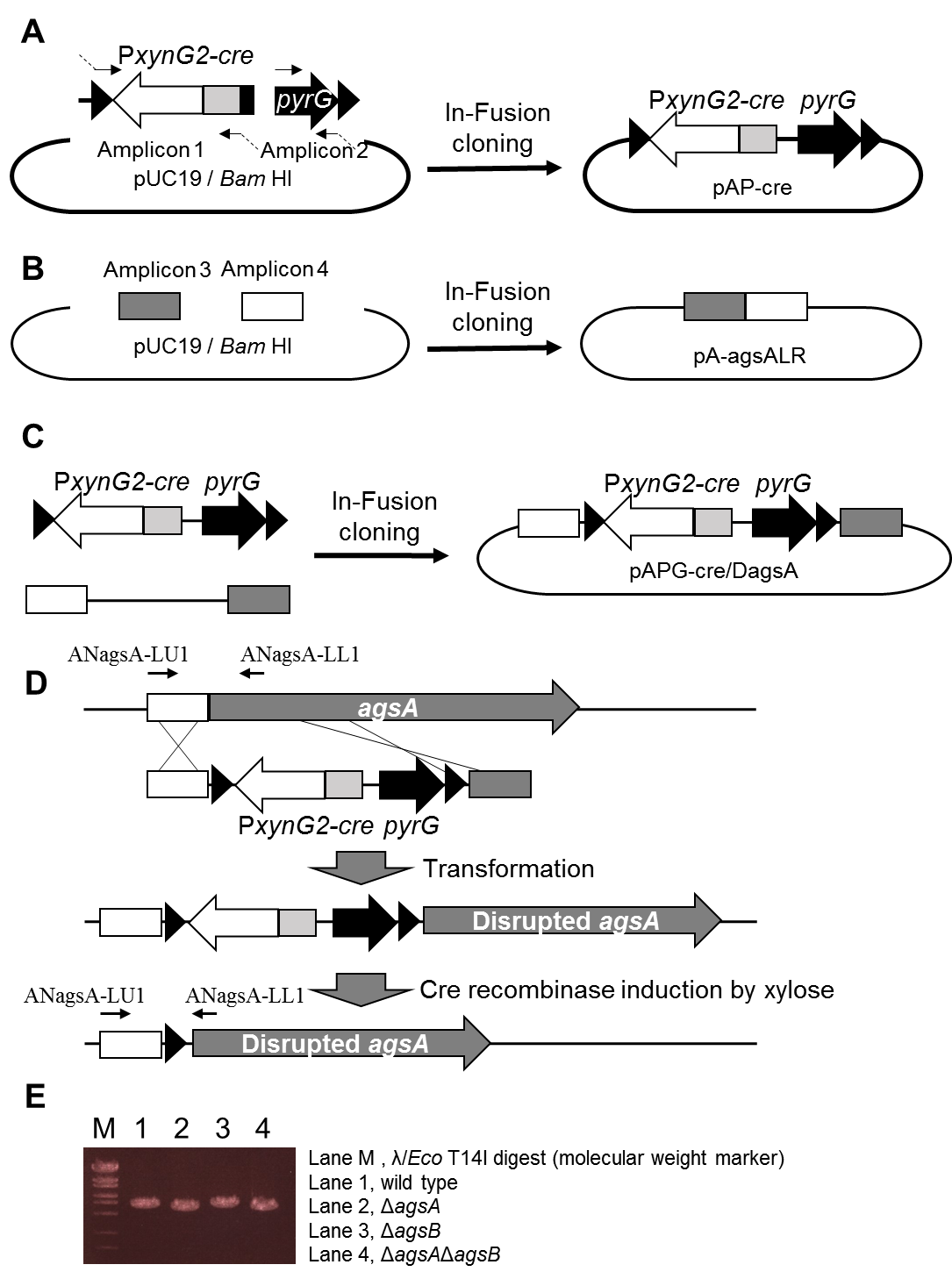


**FIGURE S1. Construction of the *agsA* disruption strain of *Aspergillus nidulans* using the Cre/mutant *loxP* marker recycling system.** (**A**) Construction of the pAP-cre plasmid. Fragments containing P*xynG2* and *Cre*, and the *pyrG* marker were amplified and then fused to *Bam*HI-digested pUC19 by using In-Fusion cloning. (**B**) Construction of the pA-agsALR plasmid. Fragments containing the right and left arms of *agsA* for *agsA* disruption were amplified and then fused to *Bam*HI-digested pUC19. (**C**) Construction of the pAPG-cre/DagsA plasmid. (**D**) Strategy for gene replacement to disrupt *agsA*. *Eco*RI-digested pAPG-cre/DagsA was used to transform the wild-type and Δ*agsB* strains. Candidate strains were isolated based on uridine and uracil requirement. Then the *Cre* gene was induced by culture on CD medium containing 1% xylose, resulting in marker cassette excision. (**E**) PCR analysis of *agsA* gene disruption.


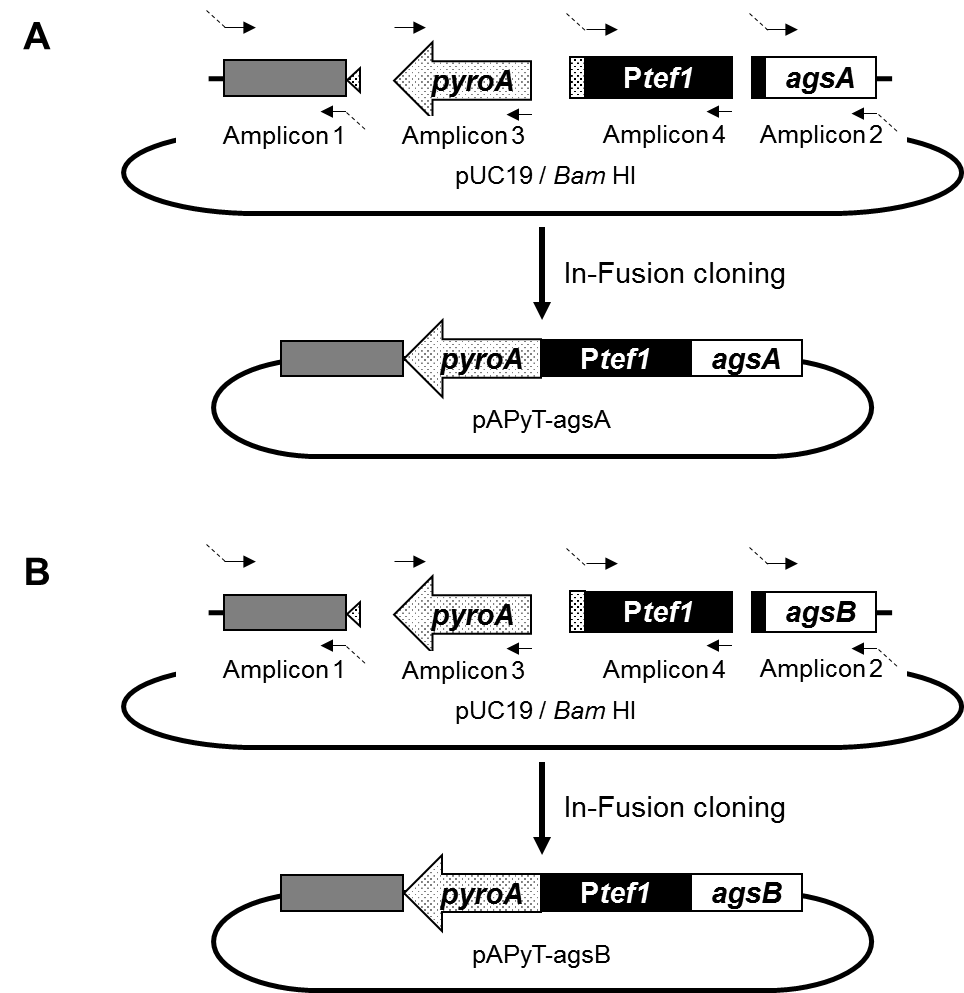


**FIGURE S2. Construction of the plasmids (A) pAPyT-agsA and (B) pAPyT-agsB for *agsA* or *agsB* overexpression.** Fragments containing the right and left arms of *agsA* or *agsB* for *agsA* or *agsB* gene replacement, the *pyroA* marker, and the *tef1* promoter (P*tef1*) for gene overexpression were amplified and then fused to *Bam*HI-digested pUC19.


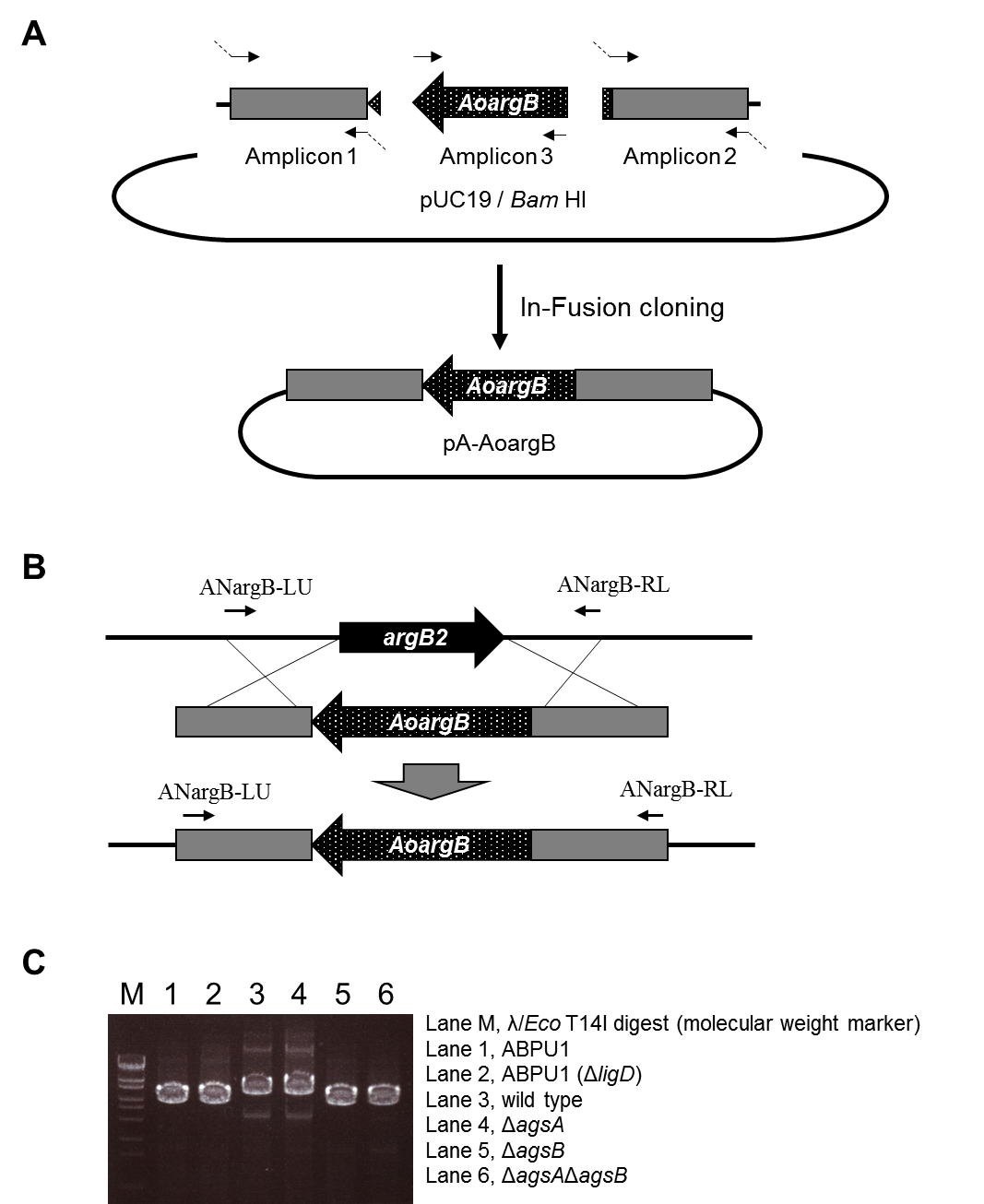


**FIGURE S3. Construction of the *argB* complementation strains of *A*. *nidulans*.** (**A**) Construction of the pA-AoargB plasmid. Fragments containing the right and left arms of the *A*. *nidulans* *argB* gene and the *argB* marker derived from *A*. *oryzae* were amplified and then fused to *Bam*HI-digested pUC19. (**B**) Strategy for replacement of the *A*. *nidulans* *argB* gene. *Not*I-digested pA-AoargB was used to transform strains requiring arginine. Candidate strains were isolated based on arginine requirement. (**C**) PCR analysis of *argB* gene complementation at the *argB* locus in *A*. *nidulans*.


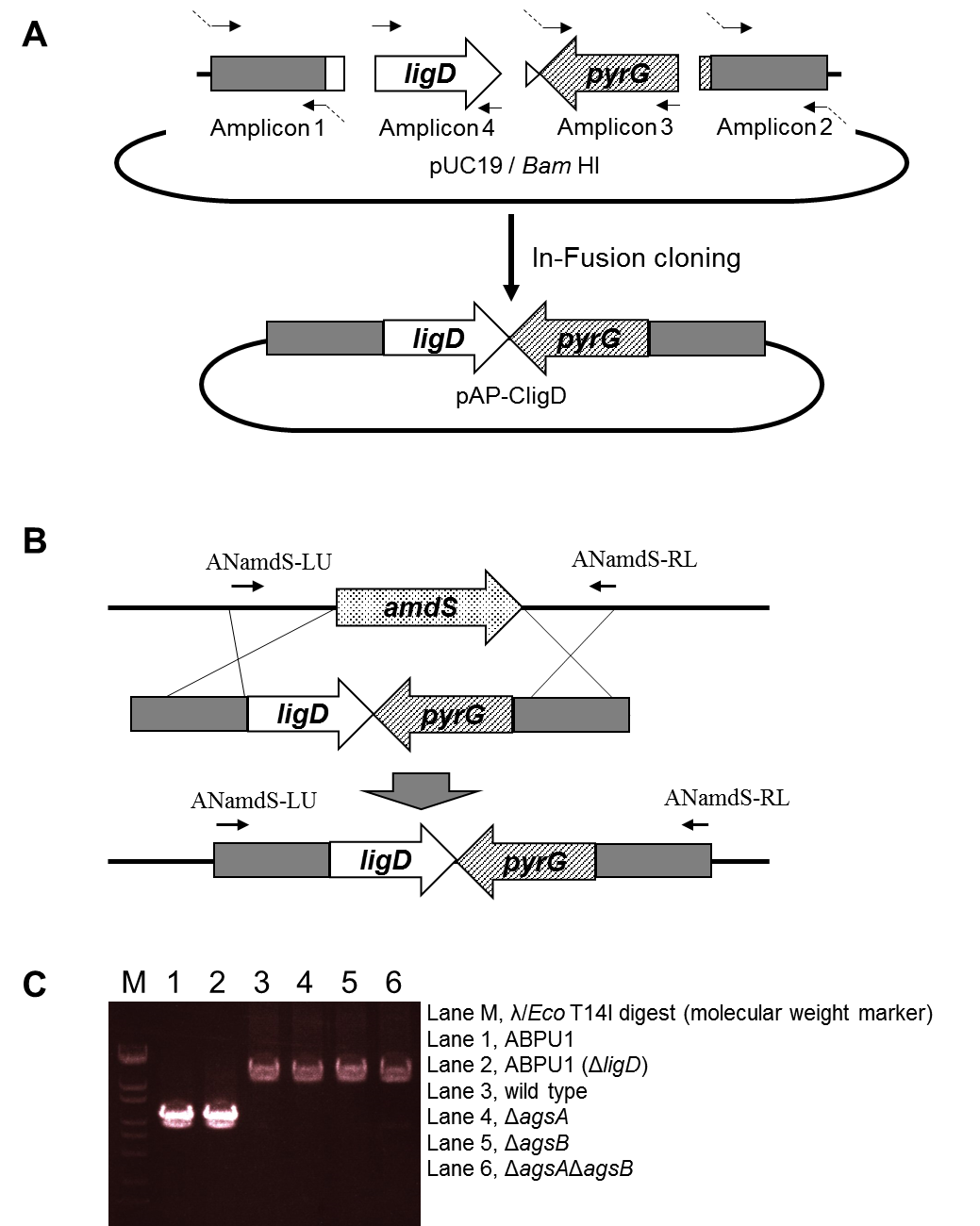


**FIGURE S4. Construction of the *ligD* complementation strains.** (**A**) Construction of the pAP-CligD plasmid. Fragments containing the right and left arms of the *A*. *nidulans* *amdS* gene, the full-length *ligD* gene, and the *pyrG* marker derived from *A*. *oryzae* were amplified and fused to *Bam*HI-digested pUC19. (**B**) Strategy for complementation of the *ligD* gene. *Not*I-digested pAP-CligD was used to transform *A*. *nidulans*. Candidate strains were isolated based on uridine and uracil requirement. (**C**) PCR analysis of *ligD* gene complementation at the *amdS* locus in *A*. *nidulans*.


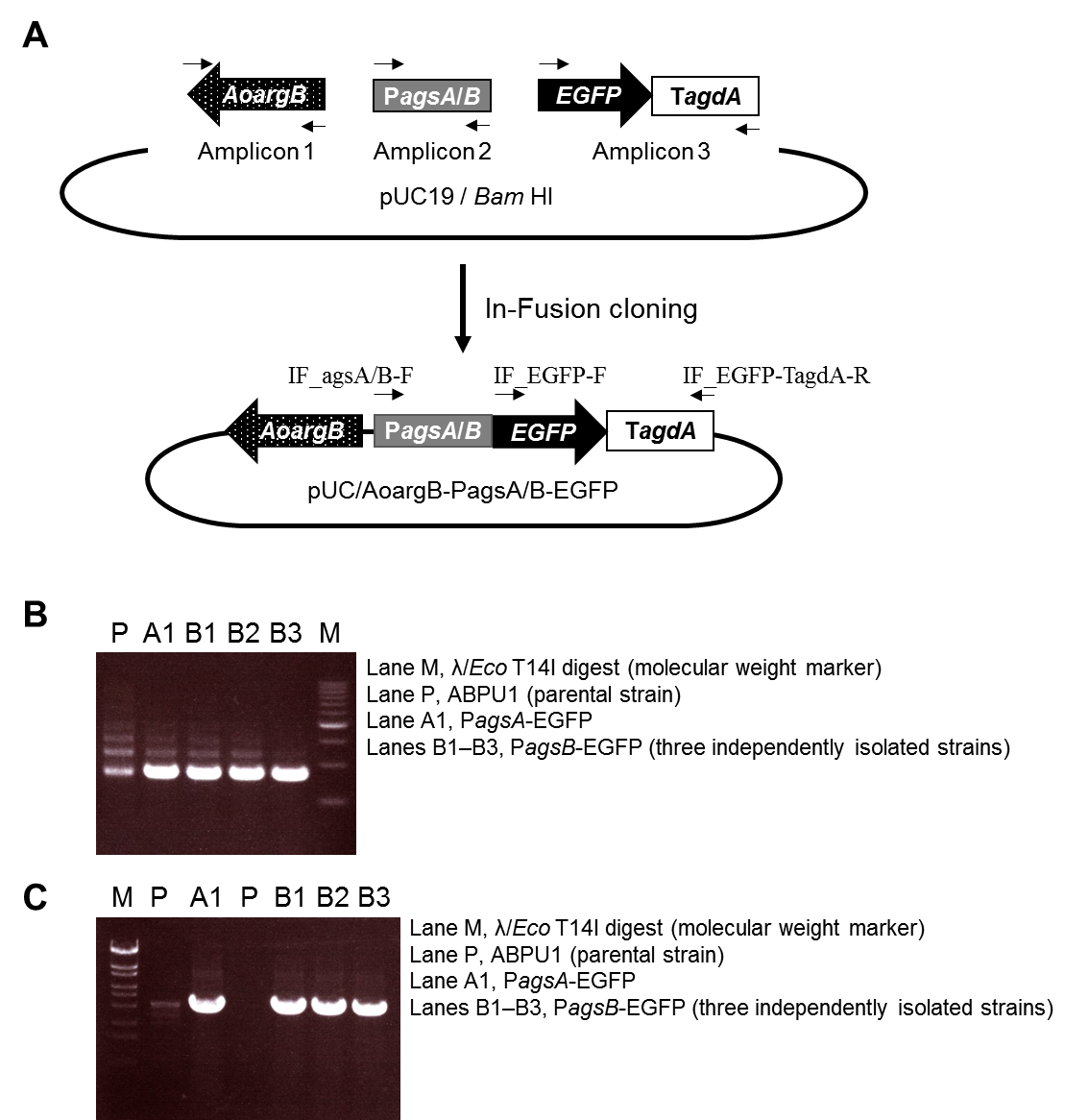


**FIGURE S5. Construction of the P*agsA*-EGFP and P*agsB*-EGFP strains of *A.* *nidulans*.** (**A**) Construction of the plasmid pUC/AoargB-PagsA/B-EGFP. Fragments containing the *argB* marker gene derived from *A*. *oryzae*, the promoter regions of *agsA* or *agsB*, and *EGFP* and the terminator region of *agdA* were amplified and fused to *Bam*HI-digested pUC19. (**B**) PCR analysis of the introduction of the *EGFP* gene into the genome. Primers IF_EGFP-F and IF_EGFP-TagdA-R were used. (**C**) PCR analysis for confirmation of the fusion of the *agsA* or *agsB* promoter region with the *EGFP* gene in the genome. Primers IF_agsA-F or IF_agsB-F and IF_EGFP-TagdA-R were used. Lane designations are as in (**B**).


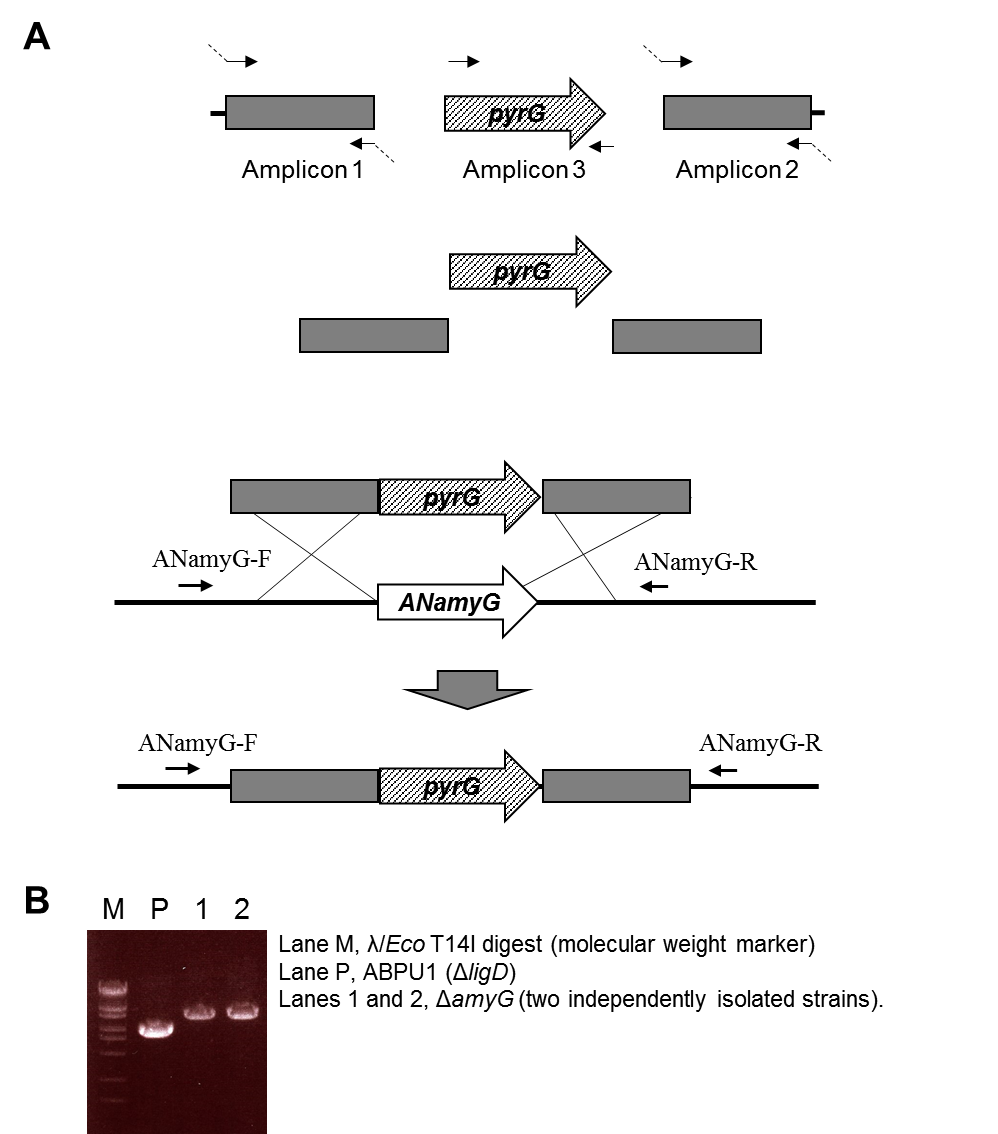


**FIGURE S6. Construction of the *amyG* disruption strain of *A.* *nidulans*.** (**A**) Disruption of the *amyG* gene. The first round of PCR was done to amplify the fragments containing the right and left arms of *amyG*, and the *pyrG* marker. The second round of PCR was done to fuse the three fragments from the first round. (**B**) PCR analysis of *amyG* gene disruption.


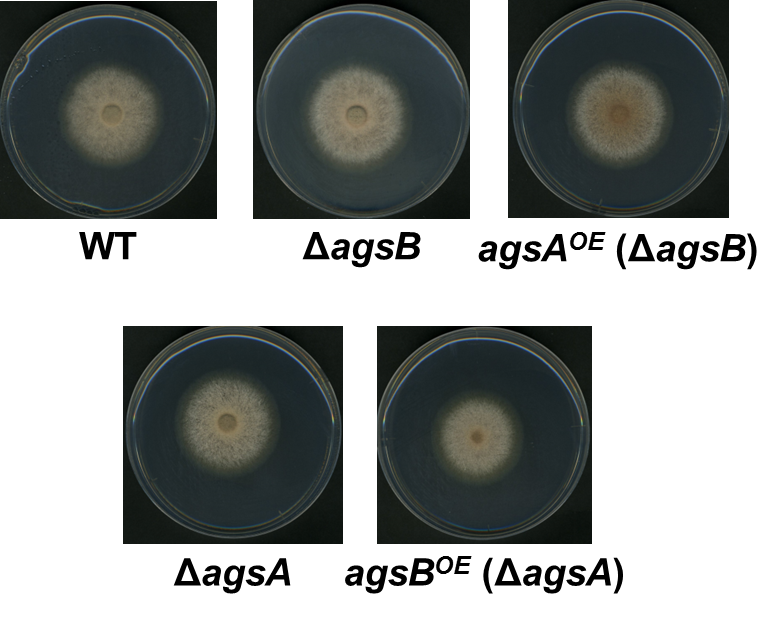


**FIGURE S7. Mycelial growth of the *agsA^OE^* and *agsB^OE^* strains on agar plates.** Conidia (1 × 10^4^) were inoculated onto CD agar medium and cultured at 37°C for 4 days.


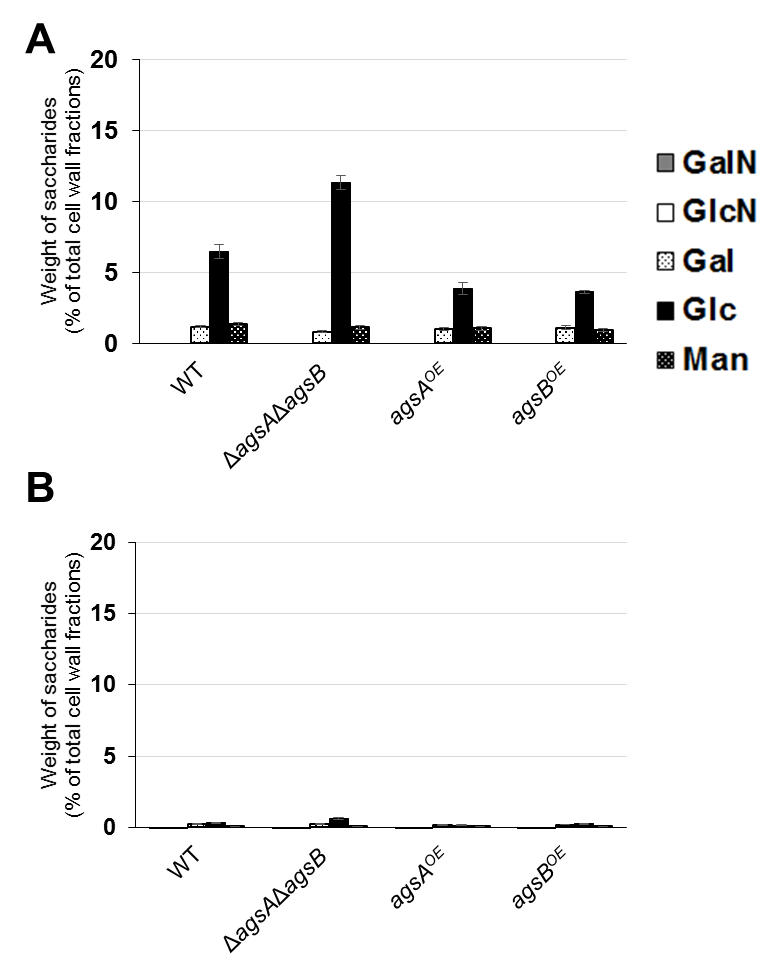


**FIGURE S8. Monosaccharide composition of the hot-water soluble (HW) and AS1 fractions from the wild-type (WT), Δ*agsA*Δ*agsB*, *agsA^OE^*, and *agsB^OE^* strains.** The strains were cultured in CD medium (160 rpm, 37°C, 24 h). The cell wall of each strain was fractionated into four fractions according to solubility in hot water at neutral pH and alkali. Monosaccharide composition of (**A**) HW and (**B**) AS1 is shown. Error bars represent the standard error of the mean calculated from three replicates. GalN, galactosamine; GlcN, glucosamine; Gal, galactose; Glc, glucose; Man, mannose.


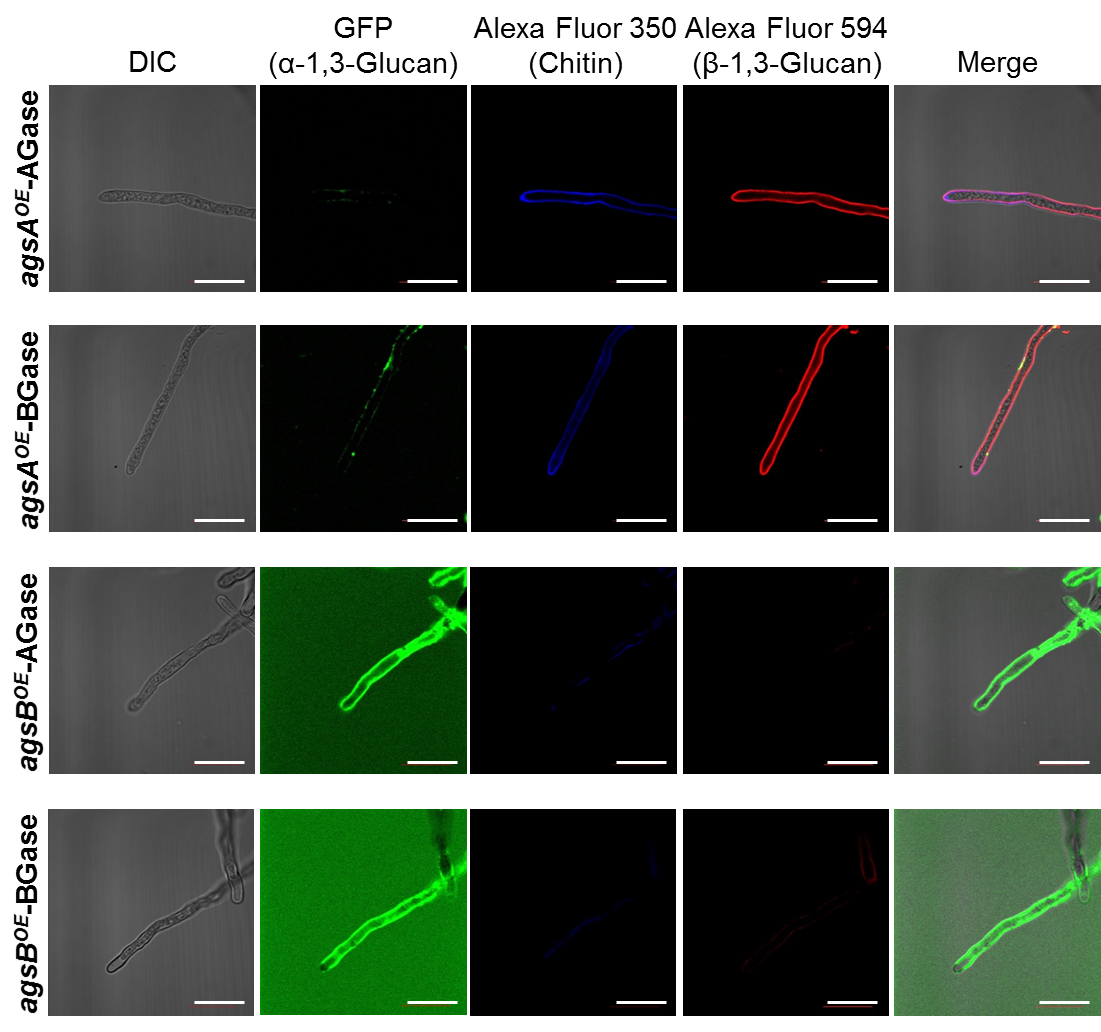


**FIGURE S9. Localization of cell wall polysaccharides without α-1,3-glucanase or β-1,3-glucanase treatment.** Vegetative hyphae cultured for 12 h were fixed and incubated for 6 h at 37°C (control for α-1,3-glucanase treatment) or 55°C (control for β-1,3-glucanase treatment). The hyphae were stained with AGBD-GFP for α-1,3-glucan, fluorophore-labeled antibody for β-1,3-glucan, and fluorophore-labeled lectin for chitin. Scale bars are 10 µm.
